# Synthesis, Molecular Recognition Study and Liquid Membrane-Based Applications of Highly Lipophilic Enantiopure Acridino-Crown Ethers

**DOI:** 10.3390/molecules25112571

**Published:** 2020-05-31

**Authors:** Ádám Golcs, Bálint Árpád Ádám, Viola Horváth, Tünde Tóth, Péter Huszthy

**Affiliations:** 1 Department of Organic Chemistry and Technology, Budapest University of Technology and Economics, Szent Gellért tér 4., H-1111 Budapest, Hungary; abalint09@gmail.com (B.Á.Á.); ttoth@mail.bme.hu (T.T.); huszthy@mail.bme.hu (P.H.); 2 MTA-BME Computation Driven Chemistry Research Group, Szent Gellért tér 4., H-1521 Budapest, Hungary; vhorvath@mail.bme.hu; 3 Institute for Energy Security and Environmental Safety, Centre for Energy Research, Konkoly-Thege Miklós út 29-33., H-1121 Budapest, Hungary

**Keywords:** chirality, ion-selective electrode, macrocycles, membrane transport, molecular recognition

## Abstract

New highly lipophilic enantiopure crown ethers containing a heterocyclic unit have been synthesized. Phase transport, UV-Vis- and fluorescence spectrophotometric investigations as well as electrochemical studies on the complexation of the new macrocycles with several amine and amino acid derivatives were also carried out. Achiral amines were used for studying the structural preference of the new macrocycles. Among the studied structural features of the guest molecules, the intermolecular *π*-*π* interaction showed the most significant effect on complexation, which made the aralkylamine-type compounds the most preferable guest molecules. The studied liquid membrane-based applications and photophysical investigations showed appreciable enantiomeric recognition toward some aralkylamine model compounds with homochiral preferences. New crown ether derivatives (*R*,*R*)-**2** and (*S*,*S*)-**2** were successfully applied as enantioselective carrier and sensor molecules.

## 1. Introduction

The synthesis of chiral macrocycles possessing an enantiomeric recognition ability has been the focus of interest since the beginning of supramolecular chemical research. The first chiral host molecule was synthesized by Stoddart and Szarek [1]. A few years later, chiral macrocycles capable of discriminating between the enantiomers of amine derivatives were also reported [2,3]. As the individual enantiomers of bioactive compounds often have completely different effects on living organisms, there is a growing demand today to isolate stereoisomers and investigate them separately in all fields of applied chemistry. Amines are very important targets for enantioselective discrimination studies because they are the common building blocks of several bioactive compounds. Numerous publications have focused on the supramolecular interaction-based stereoselective differentiation of amine compounds by chiral macrocyclic hosts, which has resulted in a number of applications using them as sensor [4,5,6,7,8,9,10], selector [11,12] and transporter [13] molecules. It is known that crown ethers can be successfully used as host molecules for protonated amine guest compounds, since the ammonium subunit can be stably coordinated by the nucleophilic heteroatoms of the macrocycle ring [14]. It was also found that the conformational rigidity of the host molecule increases the degree of enantiomeric recognition. The conformational rigidity of the macrocycles usually can be attained by incorporating a heterocyclic subunit [14]. Therefore, several macrocycles containing heteroaromatic subunits have been prepared and their enantiomeric discrimination ability toward protonated amine compounds has been thoroughly investigated. Numerous studies have been reported on crown ethers containing a pyrimidine [15,16], phenanthroline [17],triazole [18], pyridine [19,20], phenothiazine [21], phenazine [22], acridine [22] and also a phosphorus-containing aromatic subunit [23].

Acridino- and acridono-crown ethers have several advantages compared to their heterocyclic analogues. The acridine subunit makes the framework of the crown ether more rigid, resulting in increased selectivity for molecular recognition [22]. Furthermore, due to their chromogenic and fluorogenic properties, their complexing abilities can be studied by sensitive photophysical methods such as UV-Vis and fluorescence spectroscopies. Crown ethers containing an acridine subunit have been successfully used as enantioselective sensor [24,25,26,27] or selector molecules [28,29,30]. In various applications, it is desirable to incorporate a suitable substituent at position *9* of the acridine subunit to avoid its oxidation [31]. An important requirement for synthetic membrane-based applications of macrocycles is to provide high lipophilicity to increase the membrane solubility of the compounds. Thus, typically long alkyl chains are incorporated into the polyether ring of the macrocycles [32].

**Figure 1:**
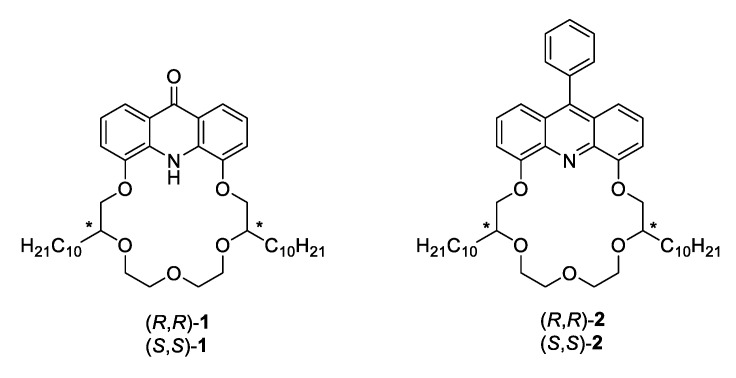
Schematics of new lipophilic enantiopure crown ethers.

In this paper, we describe the synthesis and characterization of new highly lipophilic enantiopure 18-crown-6 ether derivatives containing an acridine or an acridin-9(10*H*)-one unit (Figure 1).

The molecular recognition studies of these synthetic host molecules toward selected amines are also reported. The discriminating abilities of the new crown ethers were probed by using them in liquid membrane-based sensor and selector applications. Methods similar to the transport process presented herein may subsequently serve as the basis for enantiomeric separation or phase transfer catalytic processes, while the sensor function can be useful in determining the enantiomeric composition of known chiral amine compounds.

## 2. Results and Discussion

### 2.1. Synthesis

**Scheme 1:**
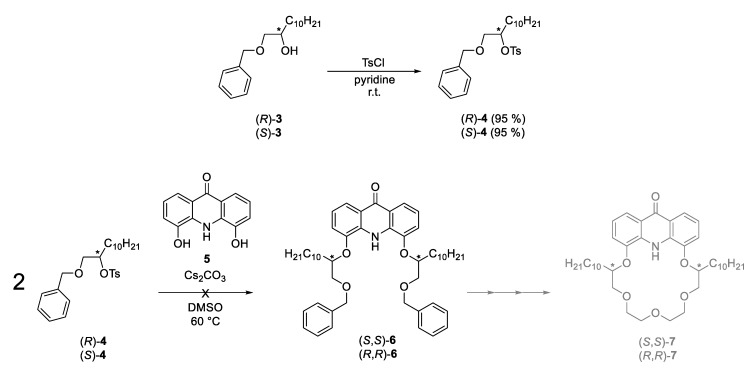
Schematics of the attempted synthesis of enantiopure lipophilic crown ethers containing an acridin-9(10*H*)-one unit.

Previous studies have demonstrated that a higher enantioselectivity can be achieved when the alkyl substituents at the chiral centres are located closer to the aromatic subunit [25,27]. For this reason, the reported [32] decyl-substituted enantiopure ethylene glycol derivatives (*R*)-**3** and (*S*)-**3** were treated with tosyl chloride in pyridine to obtain tosylates (*R*)-**4** and (*S*)-**4** in good yields, as shown in Scheme 1. Tosylates (*R*)-**4** and (*S*)-**4** were then reacted with the reported [33] acridin-9(10*H*)-onediol (**5**) in the presence of a weak base Cs_2_CO_3_ under similar conditions that are described [22,28,34,35,36] for the synthesis of similar compounds to obtain (*R*,*R*)-**6** and (*S*,*S*)-**6** derivatives, which can be used as intermediates for enantiopure crown ethers (*R*,*R*)-**7** and (*S*,*S*)-**7**.

Unfortunately, the expected products (*R*,*R*)-**6** and (*S*,*S*)-**6** could not be isolated, probably due to the steric hindrance of the long alkyl groups at the chiral centres. Because of these results, we aimed to create the asymmetric centres with a *C*-*C* bond farther from the aromatic unit.

**Scheme 2:**
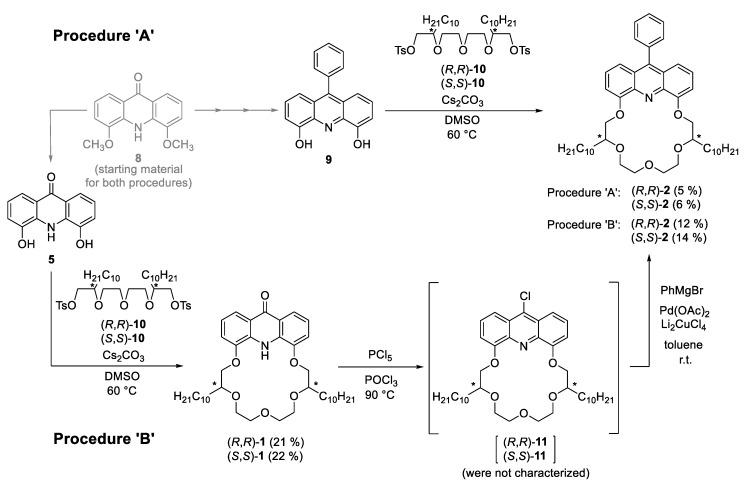
Synthesis of new lipophilic enantiopure crown ethers (*R*,*R*)-**1**, (*S*,*S*)-**1**, (*R*,*R*)-**2** and (*S*,*S*)-**2**.

The syntheses of new enantiopure didecyl-substituted crown ether ligands (*R*,*R*)-**2** and (*S*,*S*)-**2** were carried out in two ways, as outlined in Scheme 2.

In the first case (Procedure “**A**”), 9-phenylacridine-4,5-diol (**9**) was reacted with decyl-substituted enantiopure oligoethylene glycol ditosylate (*R*,*R*)-**10** or (*S*,*S*)-**10** to perform macrocyclization. The 9-phenylacridine-4,5-diol (**9**) was prepared starting from an acridin-9(10*H*)-one derivative (**8**) based on a reported procedure [31], while the tetraethylene glycol ditosylates (*R*,*R*)-**10** and (*S*,*S*)-**10** were synthesized according to an enantioselective synthesis method reported in the literature [32]. Under the applied conditions using secondary tosylates, the macrocyclization proceeds by an S*_N_*2 mechanism with a total inversion of configuration (see Scheme 1) in accordance with the previous studies [22,28,34,35,36]. In the case of primary tosylates, even by changing the reaction conditions racemization does not take place during the ring closure reaction (see Scheme 2).

In the second case (see procedure “**B**”), acridin-9(10*H*)-onediol (**5**) was used for the macrocyclization reaction in a similar manner to that described in Procedure “**A**”, using a weak base in dimethyl sulfoxide (DMSO) at 60 °C. The resulting crown compounds (*R*,*R*)-**1** and (*S*,*S*)-**1** were used for the next steps of the synthesis after purification by column chromatography. It is known that acridines can be substituted regioselectively at position *9* of their aromatic rings because of the electropositive character of the 9-*C*-atom [37]. It is also known that acridin-9(10*H*)-ones can be converted to 9-chloroacridines using phosphoryl chloride at a relatively high temperature of 90 °C [37]. The chloro-derivatives (*R*,*R*)-**11** and (*S*,*S*)-**11** were prepared by the modification of the reported method [37], applying an improved work-up procedure with an almost quantitative yield. Due to the instability of 9-chloro-acridino-compounds, the crude 9-chloro-derivatives (*R*,*R*)-**11** and (*S*,*S*)-**11** were treated with *C*-*C* coupling agents without further purification to afford the 9-phenylsubstituted macrocycles (*R*,*R*)-**2** and (*S*,*S*)-**2**, applying a reported [31] modified Kharasch-reaction in the presence of palladium and copper catalysts together.

The low yields of the *C*-*C* coupling reactions can be attributed to the instability of the 9-chloro-acridine derivatives, as acridin-9(10*H*)-one analogues were also isolated as by-products in all the coupling reactions, indicating that the transformation of the chloro-compound took place.

Previous studies have shown that the substitution with the phenyl group after macrocyclization generally results in a reduced overall yield [31]. Due to the long carbon chains of the ditosylates and the phenyl group of the acridine unit, steric hindrance effects the ring closure in both procedures “**A**” and “**B**” (see Scheme 2). Thus, only a low overall yield of each multistep reaction starting from dimethoxyacridin-9(10*H*)-one (**8**) could be obtained.

### 2.2. Membrane Transport Studies

#### 2.2.1. Investigation of the Influence of Structure on Molecular Recognition

Phase transfer catalytic processes are widely studied because they have an important role in the execution of several reactions. Phase transfer catalysts facilitate the completion of reactions by transferring materials between two immiscible phases. Similar transport processes can be accomplished by the new lipophilic crown ether derivatives reported here, which are suitable candidates for the selective transport of metal ions or protonated biogenic amines and their counterions by a passive transport mechanism driven by the concentration gradient between the phases. In addition, these crown ether derivatives may be useful hosts in the development of procedures for the separation of amines, even in extraction-based enantiomeric separation processes.

**Figure 2:**
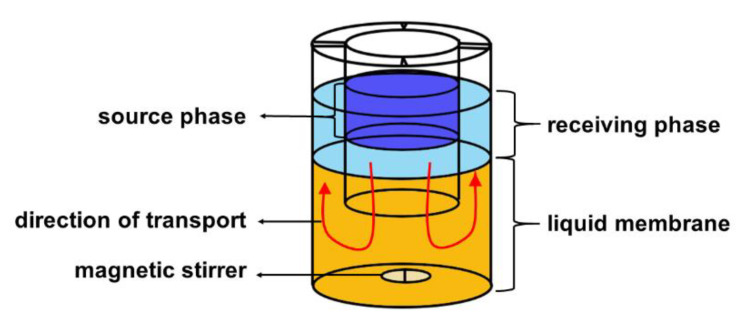
Bulk liquid membrane cell used for phase transport studies (active transfer surface: 7.07 × 10^−4^ m^2^).

The main purpose of our research was to determine the structural preference of these new crown ethers toward amines. Moreover, studies on the transport may provide valuable information for planning efficient carrier molecule-based transfer and extraction processes. For studying the liquid membrane transport, we used an apparatus shown in Figure 2 which is very similar to the transport equipment recommended in the literature [38,39,40].

**Figure 3:**
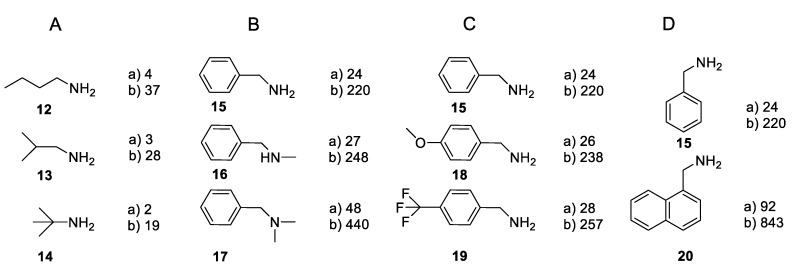
Transported amounts of amines after 20 h of transport time, measuring the ammonium salt contents of the receiving phase using crown ether (*R*,*R*)-**2** as a carrier: (a) transported rate of amines in percent (%); (b) transported quantity of amines as flux (mol/(s × m^2^) × 10^7^). The notations (**A**–**D**) indicate the grouping according to structural features: (**A**) aliphatic amines, (**B**) aralkylamines with different numbers of *N*-alkyl substituents, (**C**) aralkylamines with different *para*-substituents, (**D**) aralkylamines with different aromatic ring systems

At the beginning the source phase was an aqueous solution (2 mL) of acetic acid salts of various amines in 1 mol/L concentration, while the receiving phase was 6 mL of distilled water. The bulk liquid membrane was a solution of the crown ether in 12 mL of dichloromethane, which proved to be an appropriate solvent due to its high apolarity and density. Using an apolar membrane solvent prevents membrane leakage and allows only the carrier-mediated transport of the amines. The concentration of crown ethers in the membrane was 1 mmol/L, and thus the amines were present in more than a hundredfold excess during the transport. The transported weight-percentage value of the ammonium salt content in the receiving phase was chosen as output parameter as well as flux, since it includes information about both the transport time and the geometry of the applied apparatus. The structural preference of the crown ethers toward the amine model compounds was investigated from various aspects (Figure 3A–D). The results are summarized in Figure 3.

According to our experience, the acridono crown ethers (*R*,*R*)-**1** and (*S*,*S*)-**1** are not suitable transport molecules for amines. This is probably due to their inability to form complexes with protonated amines, a presumption which is supported by different methods in this paper below.

It can be seen that the branching of the chain in the case of aliphatic amines adversely affects the transport efficiency (see Figure 3A). In the case of amines containing an aromatic unit, the flux values increased compared to aliphatic amines. The improved transport ability compared to alkyl amines can be attributed to the *π*-*π* interaction between the aromatic moiety of the ligand and the heterocyclic unit of the host molecule. Alkylation of the amino group causes increased amounts of transported amines, which is due to increased lipophilicity (see Figure 3B). Although in this experiment higher lipophilicity was found to be favoured for transport through the apolar bulk membrane, in solution-phase studies primary amines are preferred for complexation because of the greater number of possible hydrogen bonds and for steric reasons [41]. The presence of an electron-donating or electron-withdrawing substituent at position *4* of the aromatic ring has only a negligible effect on the transport (see Figure 3C). Therefore, the degree of *π*-*π* interaction within the complex seemed to be only slightly dependent on the electropositive nature of the aromatic unit of the guest molecule. The presence of a larger aromatic system, such as a naphthyl group, proved to be the most significant of all the variations examined (see Figure 3D). This is due to the larger degree of *π*-*π* interaction within the complex. Moreover, it results in a further increase in lipophilicity.

#### 2.2.2. Enantioselective Phase Transport Studies

After studying the structural relationships of complex formation, we aimed to study the enantioselective transport of crown ethers (*R*,*R*)-**2** and (*S*,*S*)-**2**. Since the highest transport efficiency was achieved for 1-(naphthalen-1-yl)methanamine (**20**), the kinetic study of enantioselective phase transport was performed with its chiral analogue 1-(naphthalen-1-yl)ethan-1-amine (*R*)-**21** and (*S*)-**21** ((*R*)-NEA and (*S*)-NEA) and its phenyl analogue 1-phenylethan-1-amine (*R*)-**22** and (*S*)-**22** ((*R*)-PEA and (*S*)-PEA) as model compounds (see Figure 10).

**Figure 4:**
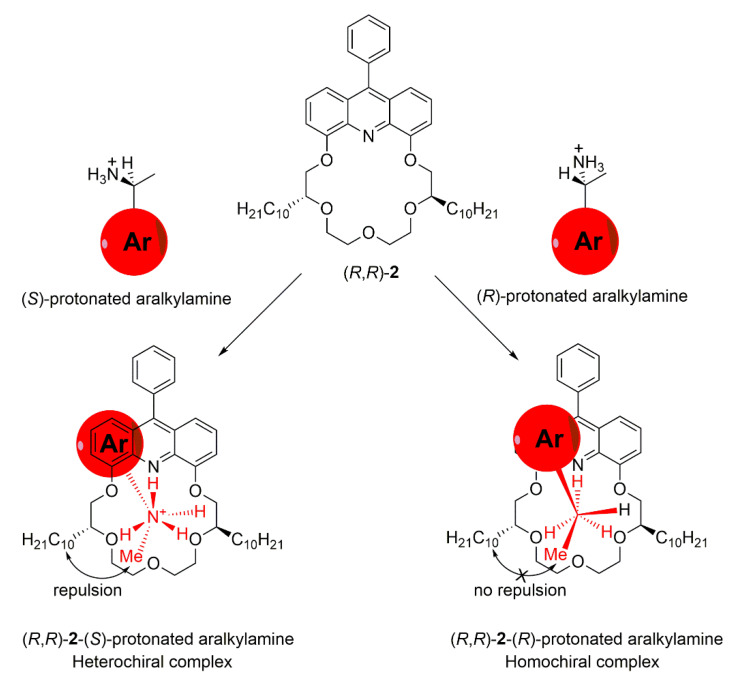
Steric repulsion causing the expected homochiral preference of the enantiopure crown ether (*R*,*R*)-**2** to aralkylamine complexation as an example.

Stereoselectivity takes place in two stages of the process. The first step is a stereoselective liquid-liquid extraction at the source phase-membrane interface. Since the membrane diffusion is carrier-mediated, in principle the stabilities of the complexes are determining. Stereoselectivity is triggered by the sterically limited positions of substituents at the chiral centres. The discrimination of the enantiomers is caused by steric repulsion, a strong nonbonding interaction, among various attractive interactions. In case of a heterochiral complex, the same orientation of the two largest groups attached to the chiral centres results in a steric repulsion. This inhibitory effect is not present in the case of the homochiral complex, and thus greater stability is expected (Figure 4).

**Figure 5:**
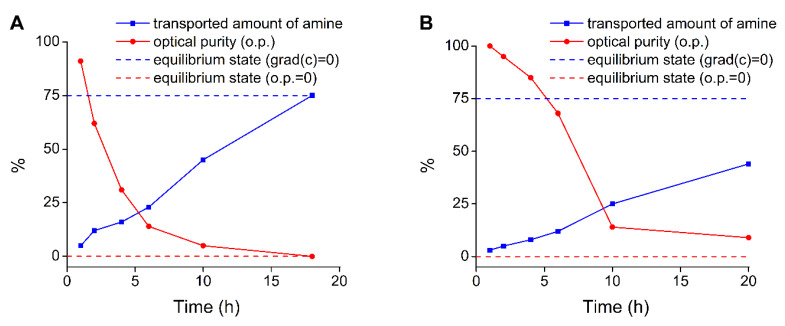
Time dependence diagrams for the transport of racemic NEA (**21**) (**A**) and racemic PEA (**22**) (**B**) using crown ether (*R*,*R*)-**2** as a carrier.

Consequently, in the following transport studies the enantiomeric excess of aralkylammonium salts estimated by optical rotation refers to the enantiomer of the homochiral preference. The transport kinetics using chiral amine model compounds NEA (**21**) and PEA (**22**) and the change in the estimated enantiomeric excess as a function of time are illustrated in Figure 5.

The greater transport efficiency for NEA (**21**) is due to the enhanced intermolecular *π*-*π* interaction between the aromatic systems and also to the increased lipophilicity, as mentioned earlier. Since none of these effects have stereochemical dependence, the increase in the transported amount of ammonium salts does not result in a higher enantioselectivity.

According to expectations, the proportion of the preferred enantiomer in the receiving phase decreased as the transport process progressed. At the initial state of the transport, the ratio of the two protonated amine enantiomers in the source phase is the same, and thus the host molecule is more likely to form a complex with the preferred enantiomer, resulting an increased flux of this stereoisomer through the membrane. After some time, the proportion of the preferred enantiomer in the source phase decreases continuously, so the probability of complex formation with the preferred stereoisomer by the host molecule also decreases, while the probability of the complexation of the less-preferred enantiomer increases in parallel. The amount of transported protonated amines increases continuously during the process, regardless of the enantiomeric composition. Therefore, the optimum time of kinetic separation should be determined by considering both the amount and the enantiomeric composition of each protonated amine in the receiving phase.

The quantitative efficacy and the selectivity of the process for the enantiomeric discrimination of optically active compounds can be combined and characterized by the following equation [42]:

(1) E=T (%)·P (%),

where *E* is a parameter to characterize the enantioselective transport efficiency, *T* stands for transported amount of ammonium salts and *P* refers to the enantiomeric proportion estimated by optical purity. The maximum value of parameter *E* is obtained at a transport time of 2 h in the case of NEA (**21**) and 6 h for PEA (**22**), which can be considered as the optimum times for the processes. In the case of NEA (**21**) the receiving phase is saturated after 18 h by reaching the equilibrium value (see Figure 5A). A couple of smaller swings in the measured values may be observed until the concentration gradient equilibrates between the phases. Since, in the case of PEA (**22**), less stereochemically independent effects take place, the decrease in the proportion of the preferred enantiomer in the receiving phase is expected to be observed later.

The type of selected counterions did not have a decisive effect either on the transported amount or on the enantioselectivity (Table 1), indicating that the carrier-mediated transport is weakly dependent on the solubility of the amine salts in the membrane. This suggests that the intermolecular *π*-*π* interaction plays a much more important role in the transport of ammonium salts as well as in complexation.

Finally, the effect of temperature was also investigated, and the results are shown in Table 2.

As expected, it was found that the increase in temperature positively influences the transported quantities and negatively affects the enantioselectivity.

These studies have shown that the crown ethers (*R*,*R*)-**2** and (*S*,*S*)-**2** are suitable carriers for enantiomeric enrichment between liquid phases, which can be useful in the enantioseparation, transport or extraction of protonated chiral aralkylamines primarily.

### 2.3. Spectrophotometric Investigation of Enantiomeric Recognition

The applicability of a macrocycle is based on the ability of the host molecule to distinguish the preferred guest molecule from a mixture of other molecules. This property can be quantified by the Δlog*K* value, which denotes the difference in the logarithms of the stability constants (*K*) of the two host-guest complexes. Spectrophotometric measurements were carried out to study the complex formation and determine the complex stability constants.

**Figure 6:**
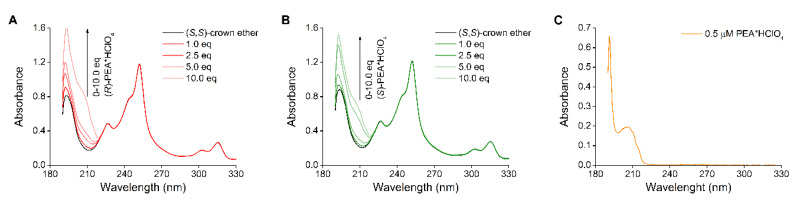
The absorption spectra of crown ether (*S*,*S*)-**1** in the presence of PEA-salts (*R*)-**22** and (*S*)-**22** (**A**,**B**) and the absorption spectrum of PEA (**22**) as a background signal (**C**).

First, the enantiomeric recognition ability of macrocycle (*S*,*S*)-**1** was investigated by its titration with the perchlorate salts of (*R*)-PEA and (*S*)-PEA (**22**) in acetonitrile using UV-Vis-spectroscopy. The resulting absorption spectra are shown in Figure 6.

In absence of complexation, no spectral change was observed in the absorption bands of the macrocycle (*S*,*S*)-**1**; the increase in absorbance below 230 nm resulted only from the background absorbance of the ammonium salt (see Figure 6C). This confirms the inability of the crown derivatives (*R*,*R*)-**1** and (*S*,*S*)-**1** to be membrane carriers of protonated amines. This may be explained by the presumption that the carbonyl group of the macrocycle is capable of forming an intermolecular hydrogen bond with the ammonium proton of the salt, thereby inhibiting complex formation. Moreover, the heteroatom of the acridin-9(10*H*)-one subunit has a reduced electron donor ability, and thus it cannot interact with a protonated guest molecule; due to the stability of the acridin-9(10*H*)-one form, tautomerism to the 9-hydroxyacridine form is unlikely to take place.

**Figure 7:**
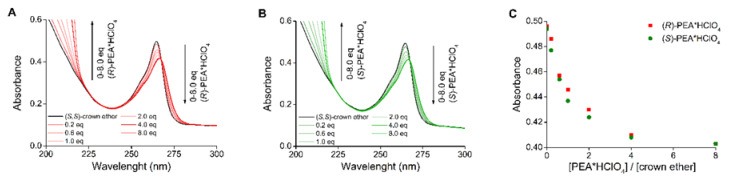
The UV absorbance changes of the crown ether (*S*,*S*)-**2** in the presence of PEA (**22**) enantiomers (**A**,**B**) and the titration curves (**C**).

The same titration was performed in the case of the crown ether (*S*,*S*)-**2**. The absorption spectra resulting from the titration with the two enantiomers of PEA (**22**) are shown in Figure 7.

It can be observed that adding more and more PEA (**22**) enantiomer causes the absorbance at 265 nm (the absorption maximum of crown ether (*S*,*S*)-**2**) to decrease steadily, indicating complex formation.

Titration curves were plotted using the absorption maximum values at 265 nm (see Figure 7C). The difference between the two titration curves is due to the difference in the stability of the diastereomeric complexes.

**Figure 8:**
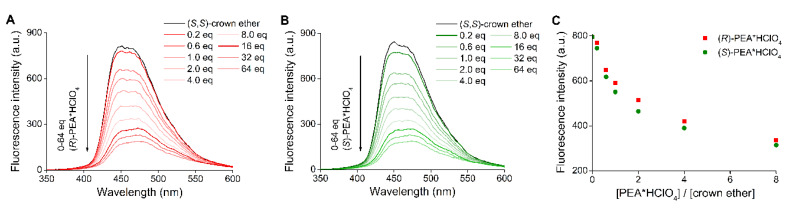
Fluorescence quenching of (*S*,*S*)-**2** upon titration with perchlorate salts of PEA enantiomers (*R*)-**22** (**A**) and (*S*)-**22** (**B**) and the titration curves (**C**).

Titration was also performed using fluorescence spectroscopy. The series of fluorescence emission spectra upon the titration of (*S*,*S*)-**2** with the two enantiomers of PEA (**22**) are shown in Figure 8A,B, and the titration curve is presented in Figure 8C. The excitation wavelength was 265 nm. The titration curves were plotted using the fluorescence emission maximum values at 465 nm. Complex formation results in a partial quenching of the fluorescence emission spectra.

**Figure 9:**
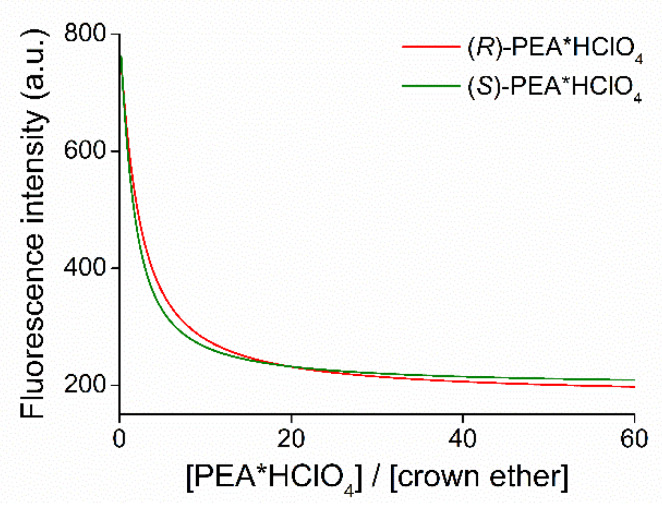
Fitted nonlinear functions for the determination of complex stability constants.

On the observed spectral changes caused by the diastereomeric complex formation of the crown ether (*S*,*S*)-**2** with the two enantiomers of PEA (**22**), global nonlinear functions were fitted over the entire wavelength range to determine the complex stability constants (log*K* values) (Figure 9). There is no overlap between the absorption and fluorescence emission spectra, and thus self-absorption and the resulting error do not need to be considered.

**Figure 10:**
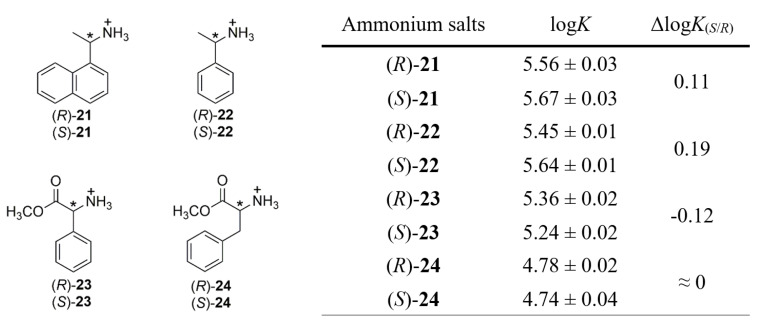
The logarithms of the stability constants (log*K* values) for the complexes of crown ether (*S*,*S*)-**2** with the enantiomers of different primary ammonium salts and their differences (Δlog*K*_(*S*/*R*)_ values), expressing the degree of enantiomeric recognition.

The complex stability constants were determined by the method described in Section 4.4 of the Experimental section (Equation (2)). According to our calculations, 1:1 crown ether:ammonium ion complex stoichiometry is preferred. The stability constants of crown ether (*S*,*S*)-**2**–ammonium perchlorate enantiomer complexes were determined for three other primary ammonium perchlorate model compounds besides PEA (**22**). The results are summarized in Figure 10.

There is an appreciable difference between the stability constants of some complexes formed with the enantiomers of ammonium salts **21**–**23**, indicating enantiomeric discrimination. The degree of enantioselectivity is expressed by the value of Δlog*K*_(*S*/*R*)_. In accordance with the observations of transport experiments, homochiral preference is observed, and thus the (*S*,*S*)-crown ether forms a more stable complex with the (*S*)-enantiomer of the protonated amine. An exception to this is the phenylglycine methyl esters (PGME) (*R*)-**23** and (*S*)-**23**, which can be explained by nomenclature rules: the preferred configuration of the guest molecule can be determined by the opposite stereo-descriptor due to the presence of an ester group. In the case of the phenylalanine methyl esters (PAME) (*R*)-**24** and (*S*)-**24**, there is no enantiomeric discrimination, which may be explained by the fact that the chiral centre is not close enough to the aromatic moiety, and thus the *π*-*π* interaction cannot sufficiently fix the guest molecule inside the complex for expressing steric repulsion. From the results, it can be generalized that the enantiomers of protonated primary amines or amino acid derivatives with an aromatic unit and a chiral centre directly attached to the *π*-system form complexes with (*S*,*S*)-**2** with different stabilities. Therefore, the new crown ether (*S*,*S*)-**2** or its enantiomer is a suitable optical enantiosensor molecule and can also be applied in other enantioselective sensor devices for distinguishing protonated amine derivatives with the mentioned structural features.

### 2.4. Development of Enantioselective Potentiometric Sensors

**Figure 11:**
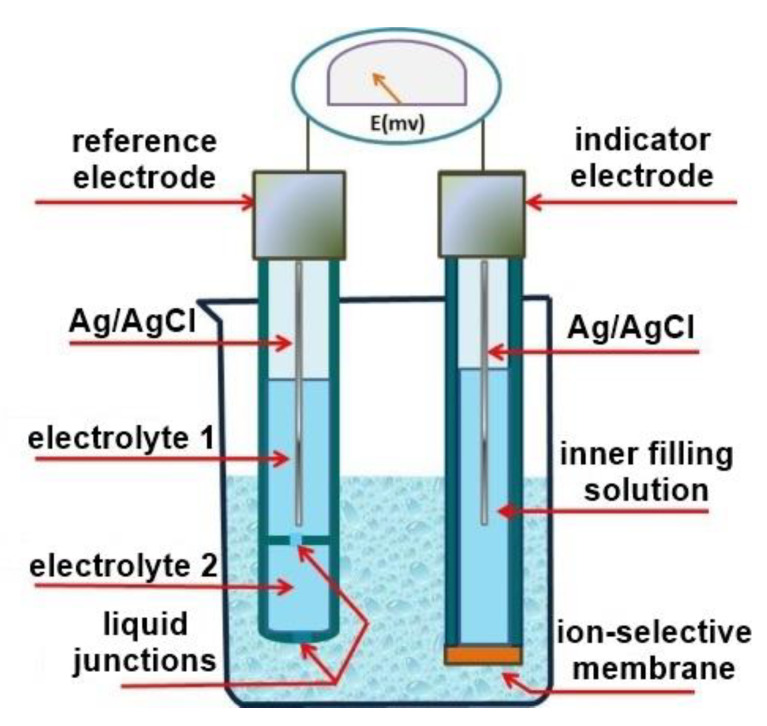
Illustration [43] of the potentiometric cell used for the present electrochemical studies.

From the aspect of signalization, potentiometric electrodes are one of the most popular chemical sensors; they are easy to use, low-cost, miniature devices. They provide the analytical information as an electrical signal, which is advantageous for instrument development. A widely used type of electrochemical sensor is the synthetic neutral ionophore-based ion-selective membrane electrode shown in Figure 11, which can contain a crown ether immobilized in a plasticized PVC (polyvinyl chloride) membrane. The potentiometric signal is obtained as the electromotive force (EMF) of the measuring cell formed by the reference electrode and the ion-selective indicator electrode.

**Figure 12:**
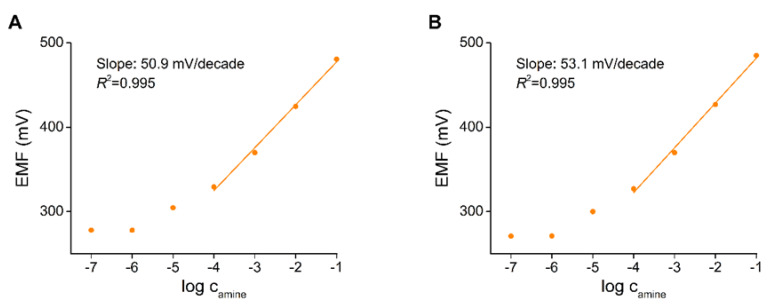
Calibration curves of the membrane electrodes containing the crown ether (*R*,*R*)-**1** (**A**) or (*R*,*R*)-**2** (**B**) as an ionophore with the hydrochloride salt of racemic NEA (**21**).

Following the incorporation of macrocycles **1** and **2** into the bulk membranes, the indicator electrodes were calibrated using a dilution series of racemic protonated amine model compounds **21**–**24** in aqueous solutions. A regression line was fitted to the linear range of the calibration curve to determine the slope of the response function. The curve obtained for the crown ether (*R*,*R*)-**1** is shown in Figure 12A, while the calibration curve for the macrocycle (*R*,*R*)-**2** is shown in Figure 12B.

The electrode gives a near-Nernstian response to protonated NEA (**21**), with an average slope of 50.9 mV/decade in the case of crown ether (*R*,*R*)-**1** and 53.1 mV/decade in the case of macrocycle (*R*,*R*)-**2** in the concentration range of 10^−1^–10^−4^ M and with a potentiometric response time of 40 s. The calibration curve is linear between the 10^−1^ and 10^−4^ M concentrations. Below 10^−4^ M, a decreased slope of the calibration curve was observed. The detection limit is approximately 1 × 10^−5^ M.

The sensor can be used for analysis in aqueous media between pH 4 and 7 using maximum 20% of organic solvent content (investigated by adding either methanol or acetone). The protonation of the basic nitrogen of the sensor molecule takes place below pH 4, while in alkaline media the precipitation of amines from water can take place. Large amounts of organic solvent in the water samples result in a shift in the response and may cause membrane damage. Although higher membrane affinities of the amine salts with more lipophilic counterions would be expected due to the increased Hoffmeister-type selectivity, the use of different anions (investigated with acetate and chloride salts) did not influence the response signal. We have found that the potentiometric selectivity coefficients for protonated aliphatic amine derivatives (investigated with isopropyl amine and isobutyl amine) are more than 6 orders of magnitude lower than for aromatic amines. As aliphatic protonated amines show a lower affinity for these apolar membranes and are less preferred by the ionophores, they do not give an electrode response comparable to protonated aralkylamines. As protonated aralkylamine derivatives are much preferred for complexation, they can selectively be detected in the presence of aliphatic impurities.

**Figure 13:**
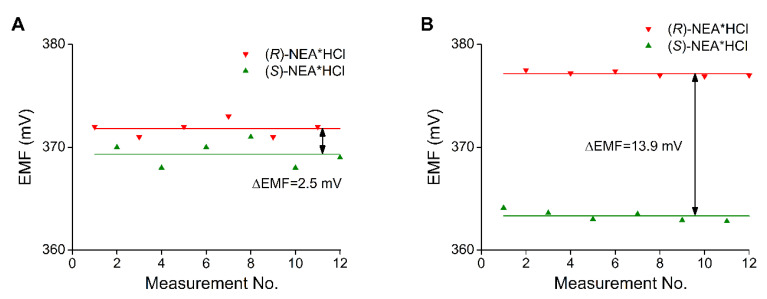
Measured electromotive force (EMF) values of ionophore (*R*,*R*)-**1** (**A**) or (*R*,*R*)-**2** (**B**) containing ion-selective membranes in the presence of enantiomers of NEA (**21**).

The enantioselectivities of ligands **1** and **2** for the enantiomers of protonated aralkylamine derivatives **21**–**24** were assessed by alternately immersing the electrode in aqueous 10^−3^ M (*R*)- and (*S*)-aralkylammonium chloride solutions and recording the electrochemical responses (EMF). The results for NEA (**21**) are shown in Figure 13.

In accordance with the membrane transport and spectrophotometric investigations, the electrode containing (*R*,*R*)-**1** was not able to distinguish the enantiomers of NEA (**21**), while the electrode containing (*R*,*R*)-**2** showed a marked difference in potential response toward the enantiomers with a homochiral preference. The differences in the EMF values were used to calculate the potentiometric selectivity factors ($K_{\left(S/R\right)}^{\mathrm{pot}}$, $\frac{\beta_{RL}^{w}}{\beta_{SL}^{w}}$). The selectivity coefficient ideally gives a good approximation of the ratio of the stability constant of the two diastereomeric complexes formed by crown ethers and the different enantiomers of protonated amines [44]. In this case, the potentiometric selectivity factors will be comparable to the selectivity constants obtained in spectrophotometric determinations. The selectivity factors of ligands (*R*,*R*)-**1** and (*R*,*R*)-**2** assessed by potentiometry for the enantiomers of protonated amine derivatives **21**–**24** are shown in Table 3.

Electrochemical measurements confirm the results of the transport experiments and those of the spectroscopic examinations. There are no significant differences among the selectivity values determined by the various methods. Only small divergences occur due to the application of different solvents or to differences in the applied methods. In the case of crown ether **1**, no significant complex formation occurred with the protonated amine derivatives, which may have a structural cause discussed previously. Only for NEA (**21**) was a slightly detectable difference in enantioselectivity observed, which could be quantified by potentiometry. In the case of macrocycle **2**, only the enantiomers of PAME (**24**) could not be discriminated well. For the other three aralkylammonium salts **21**–**23**, there are significant differences in the stability constants of the complexes formed with the enantiomers.

## 3. Conclusions

We reported the synthesis and characterization of new enantiopure highly lipophilic acridono- and acridino-crown ether derivatives, including the synthetic difficulties encountered during the preparation of the new compounds. The new macrocycles proved to be useful candidates as sensor, selector and transporter molecules in various liquid membrane-based applications.

The molecular recognition of the new macrocycles and related structural preferences toward several protonated amine guest molecules were studied by liquid membrane phase transport. We have shown that the complexation of protonated aralkylamine derivatives is preferable. A comparison of the crown ethers showed that macrocycles containing a heteroaromatic unit in their acridine form are more effective in the complexation of protonated amines than their acridin-9(10*H*)-one analogues. The instability of the acridine subunit was eliminated by the incorporation of a phenyl substituent at position *9*.

The enantiomeric recognition of the chiral host molecules was studied by membrane transport, solution-phase UV-Vis and fluorescence spectroscopies, as well as by electrochemical methods. Effective enantiomeric recognition was observed when the protonated amine or amino acid derivative had a chiral centre as close as possible to its aromatic moiety. In the context of these studies, we have found structural and physicochemical relationships which may be useful in the design of membrane-based applications of similar host molecules.

For quantifying the enantioselectivity toward chiral protonated amines and amino acid derivatives, the new lipophilic crown ethers were investigated using four model compounds by photophysical methods and also by potentiometry. Similar results were obtained by the different methods. The reported ion-selective membrane electrode proved to be effective in determining the enantiomeric composition of the protonated aralkylamine model compounds. The latter analytical method is fast and requires simple instrumentation and a very small amount (1 mg) of sensor molecule.

In the future, we plan to develop an enantiomeric enrichment process based on separation by an (*R*,*R*)-**2** or (*S*,*S*)-**2**-containing liquid membrane, which is expected to allow a high throughput enantiomeric enrichment of bioactive amines on a milligram scale.

## 4. Experimental

### 4.1. General

The starting materials and reagents were purchased from Sigma-Aldrich Corporation (USA, owned by Merck) and used without further purification unless otherwise noted. Solvents were dried and purified according to well established methods [45]. Silica Gel 60 F_254_ (Merck, Darmstadt, Germany) and aluminium oxide 60 F_254_ neutral type E (Merck, Germany) plates were used for thin-layer chromatography (TLC). All the reactions were monitored by TLC and visualized by UV lamp. Aluminium oxide (neutral, activated, Brockman I) and Silica Gel 60 (70–230 mesh, Merck) were used for column chromatography. Purifications by preparative thin-layer chromatography (PTLC) were carried out using Silica gel 60 F_254_ (Merck, Germany) plates of 2 mm layer thickness (art No.: 1.05744) or aluminium oxide 60 F_254_ neutral type E (Merck, Germany) plates of 0.25 mm layer thickness (art No.: 1.05727). The ratios of solvents for the eluents are given in volumes (mL/mL). Evaporations were carried out under a reduced pressure unless otherwise stated.

The new compounds were characterized by their physical constants such as melting point, optical rotation, thin-layer chromatography retention factor (*R*_f_), infrared, ^1^H-NMR and ^13^C-NMR spectroscopies and MS or HRMS spectrometry. ^1^H-NMR and ^13^C-NMR spectra of the starting materials and the new compounds are available in Supplementary Materials from Figure S1 to Figure S14. Spectroscopic measurements were carried out at room temperature. The melting points were taken on a Boetius micro-melting point apparatus and are uncorrected. The infrared spectra were recorded on a Bruker Alpha-T FT-IR spectrometer (Bruker Corporation, Billerica, MA, USA) using KBr pastilles. Optical rotations were taken on a Perkin-Elmer 241 polarimeter (PerkinElmer Inc., Waltham, MA, USA) that was calibrated by measuring the optical rotations of both the enantiomers of menthol. The NMR spectra were recorded on a Bruker 300 Avance spectrometer (Bruker Corporation, USA; at 300 MHz for ^1^H and at 75.5 MHz for ^13^C spectra). Mass spectra were recorded on an Agilent-1200 Quadrupole LC/MS Instrument (Agilent Technologies, Inc., Santa Clara, CA, USA) using electrospray ionization. HRMS analyses were performed on a Thermo Velos Pro Orbitrap Elite (Thermo Fisher Scientific, Darmstadt, Germany) system. The ionization method was ESI (electrospray ionization) and operated in positive ion mode. The protonated molecular ion peaks were fragmented by CID (collision-induced dissociation technique) at a normalized collision energy of 35%–45%. The samples were dissolved in methanol. Data acquisition and analysis were accomplished with Xcalibur software version 2.2 (Thermo Fisher Scientific, Germany). The UV-Vis spectra were recorded on a UNICAM UV4-100 spectrophotometer controlled by VIZION 3.4 software (ATI UNICAM, Knutsford, UK). The fluorescence emission spectra were recorded on a Perkin-Elmer LS 50B luminescent spectrometer (PerkinElmer Inc., USA) and corrected by FL Winlab 3.0 spectrometer software (PerkinElmer Inc., USA). Quartz cuvettes with a path length of 1 cm were used in all cases. Elemental analyses were performed in the Microanalytical Laboratory of the Department of Organic Chemistry, Institute for Chemistry, L. Eötvös University, Budapest, Hungary. Chiral chromatography was performed on a VWR Hitachi Elite (Hitachi Ltd., Tokyo, Japan) HPLC system, involving a Diode Array Detector L-2450, LaChrom L-2310 pump, L-2200 autosampler, and L-2300 column oven, using a Reprosil Chiral MIA HPLC column (100 × 4.6 mm). Isocratic elution was applied with a solvent system of a 1:9 mixture of isopropanol/CH_3_CN + 0.1% trifluoroacetic acid and applying a flow rate of 0.4 mL/min at 20 °C. UV absorbance detection was carried out at 280 nm. For the determination of enantiomeric purity of crown compounds, a mixture containing both of the enantiomers with an enantiomeric ratio of (*R*,*R*):(*S*,*S*) = 1:2 was measured. Chromatograms for new enantiopure crown compounds are available in Supplementary Materials from Figure S15 to Figure S16.

### 4.2. Synthesis of New Compounds

#### 4.2.1. (2*R*)-1-(Benzyloxy)dodecan-2-yl-4-Methylbenzene-1-Sulfonate [(*R*)-**4**]: See Scheme 1

Enantiopure monobenzyl-monohydroxy compound (*R*)-**3** (2.00 g, 6.84 mmol) was dissolved in pyridine (10 mL), and a solution of tosyl chloride (1.96 g, 10.26 mmol) in pyridine (10 mL) was added dropwise to the stirred solution of the hydroxy compound. The reaction mixture was stirred at room temperature for 2 days. The solvent was removed and the residue was dissolved in a mixture of ethyl acetate (100 mL) and water (200 mL). The phases were shaken well and separated. The pH of the aqueous phase was adjusted to 7 with dilute hydrochloric acid (10 m/m%). The aqueous phase was extracted with ethyl acetate (3 × 100 mL). The combined organic phase was extracted with saturated aqueous sodium chloride solution (100 mL), dried over magnesium sulphate and filtered and the solvent was removed. The crude product was purified by column chromatography on silica gel using an ethyl acetate/hexane (1:10) mixture as an eluent to give (*R*)-**4** (2.90 g, 95%) as a colourless oil.

*R*_f_ = 0.65 (silica gel TLC, ethyl acetate/hexane 1:4). ${\left[\alpha \right]}_{D}^{25}$ = +5.76 (c = 1.02, CH_2_Cl_2_). ^1^H-NMR (CDCl_3_): δ [ppm]: 0.90 (t, *J* = 6.5 Hz, 3H, CH_3_ group located at the end of the decyl chain); 1.10–1.39 (m, 18H, CH_2_ groups of the decyl chain); 2.43 (s, 3H, *para*-CH_3_ of tosyl group); 3.47–3.60 (m, 2H, CH_2_ group attached to the benzyloxy group); 4.37–4.50 (m, 2H, benzyl CH_2_); 4.66 (p, *J* = 5.3 Hz, 1H, CH group attached to the decyl chain); 7.20–7.27 (m, 3H, aromatic protons at the meta and para positions of the benzyl group); 7.29–7.41 (m, 4H, aromatic protons at the ortho positions of the benzyl group and the aromatic ring of the tosyl group); 7.80 (d, *J* = 8.3 Hz, 2H, the other two aromatic protons of the tosyl group). ^13^C-NMR (CDCl_3_): δ [ppm]: 14.33 (CH_3_ group located at the end of the decyl chain); 21.82 (para-CH_3_ of the tosyl group); 22.90 (CH_2_ group of the decyl chain); 24.95 (CH_2_ group of the decyl chain); 29.44 (CH_2_ group of the decyl chain); 29.53 (CH_2_ group of the decyl chain); 29.58 (CH_2_ group of the decyl chain); 29.69 (CH_2_ group of the decyl chain); 29.79 (CH_2_ group of the decyl chain); 31.78 (CH_2_ group of the decyl chain); 32.12 (CH_2_ group of the decyl chain); 71.14 (CH_2_ group attached to the benzyloxy group); 73.47 (benzyl CH_2_); 82.18 (CH group attached to the decyl chain); 127.81 (ArCH); 127.88 (ArCH); 128.05 (ArCH); 128.54 (ArCH); 129.75 (ArCH); 134.61 (ArC); 137.97 (ArC); 144.54 (ArC). IR: ν_max_ [cm^−1^]: 2923, 2853, 1597, 1454, 1362, 1188, 1174, 1097, 907, 813, 735, 697, 665, 554. HRMS: *m*/*z* = [MH^+^]: 447.25158, [M]: 446.24908. Anal. Calcd. for C_26_H_38_O_4_S: C 69.92; H 8.58; S 7.18. Found: C 69.73; H 8.56; S 7.20.

#### 4.2.2. (2*S*)-1-(Benzyloxy)dodecan-2-yl-4-Methylbenzene-1-Sulfonate [(*S*)-**4**]: See Scheme 1

Tosylate (*S*)-**4** was prepared in the same way as described above for (*R*)-**4**, starting from monohydroxy compound (*S*)-**3** (2.00 g, 6.84 mmol). Yield: 2.92 g (95 %). ${\left[\alpha \right]}_{D}^{25}$ = −6.01 (c = 1.16, CH_2_Cl_2_). The other physical and spectroscopic data of (*S*)-**4** concurred with those of (*R*)-**4**.

#### 4.2.3. (8*R*,16*R*)-8,16-Bis(decyl)-6,9,12,15,18-Pentaoxa-25-Azatetracyclo [2 1.3.1.0^5,26^.0^19,24^]heptacosa-1,3,5(26),19,21,23-Hexaen-27-one [(*R*,*R*)-**1**]: See Scheme 2

4,5-Dihydroxyacridin-9(10*H*)-on (**5**) (1.23 g, 5.42 mmol) and caesium carbonate (8.83 g, 27.10 mmol) were stirred in 100 mL of pure and dry DMSO. The mixture was stirred under argon atmosphere at room temperature for 15 min. Then, the enantiopure glycol derivative (*R*,*R*)-**10** (5.11 g, 6.53 mmol) in 100 mL of dry and pure DMSO was added dropwise to the stirred mixture. After addition, the reaction mixture was raised to 60 °C and stirred at this temperature for 1 week. Water (500 mL) was added to the mixture and it was extracted with ethyl acetate (3 × 500 mL). The combined organic phase was extracted with water (6 × 500 mL) and then with saturated aqueous sodium chloride solution (2 × 500 mL) to remove most of the DMSO. The organic phase was dried over magnesium sulphate and filtered and the solvent was removed. The crude product was purified by column chromatography on neutral aluminium oxide using an ethyl acetate/hexane (1:5) mixture as an eluent. The product needed further purification by PTLC on aluminium oxide using a toluene/ethanol (200:1) mixture as an eluent to give (*R*,*R*)-**1** (758 mg, 21%) as yellow crystals.

M.p. = 85 °C. *R*_f_ = 0.75 (silica gel TLC, methanol/dichloromethane 1:20). ${\left[\alpha \right]}_{D}^{25}$ = −23.15 (c = 0.44, CH_2_Cl_2_). ^1^H-NMR (CDCl_3_): δ [ppm]: 0.89 (t, *J* = 6.5 Hz, 6H, CH_3_ groups located at the end of the decyl chains); 1.04–1.70 (m, 36H, CH_2_ groups of the decyl chains); 3.28–4.38 (m, 14H, CH_2_ groups of the macrocyclic ring); 7.07 (d, *J* = 7.4 Hz, 1H, ArCH of the heterocyclic moiety); 7.18 (t, *J* = 8.0 Hz, 2H, ArCH groups of the heterocyclic moiety); 7.35 (d, *J* = 8.1 Hz, 1H, ArCH of the heterocyclic moiety); 7.80 (d, *J* = 8.1 Hz, 1H, ArCH of the heterocyclic moiety near the attaching macroring), 8.07 (d, *J* = 8.1 Hz, 1H, ArCH of the heterocyclic moiety near the attaching macroring); 9.31 (s, broad, 1H, NH of the heterocyclic moiety). ^13^C-NMR (CDCl_3_): δ [ppm]: 14.14 (CH_3_ groups located at the end of the decyl chains); 22.72 (CH_2_ of decyl chains); 25.60 (CH_2_ of decyl chains); 29.37 (CH_2_ of decyl chains); 29.67 (CH_2_ of decyl chains); 29.84 (CH_2_ of decyl chains); 31.58 (CH_2_ of decyl chains); 31.94 (CH_2_ of decyl chains); 70.26 (CH_2_ of the macrocyclic ring); 71.07 (CH_2_ of the macrocyclic ring); 72.01 (CH groups attached to the decyl chains); 77.90 (CH_2_ groups of the macroring near the heterocyclic moiety); 112.03 (ArCH of the heterocyclic moiety); 118.52 (ArCH of the heterocyclic moiety); 120.72 (ArCH of the heterocyclic moiety); 122.14 (ArCH of the heterocyclic moiety); 127.99 (ArCH of the heterocyclic moiety); 129.85 (ArCH of the heterocyclic moiety); 131.41 (ArCH of the heterocyclic moiety); 146.63 (ArC groups of the heterocyclic moiety attached to the macroring); 177.95 (C=O carbon of the heterocyclic moiety). IR: ν_max_ [cm^−1^]: 3425, 2923, 2853, 1736, 1624, 1610, 1531, 1490, 1466, 1421, 1369, 1339, 1265, 1225, 1178, 1116, 1092, 803, 747. HRMS: *m*/*z* = [MH^+^]: 666.46814, [M]: 665.46554. Anal. Calcd. for C_41_H_63_NO_6_: C 73.95; H 9.54; N 2.10. Found: C 74.20; H 9.52; N 2.15. The enantiomeric purity was determined by chiral HPLC and found to be >97%.

#### 4.2.4. (8*S*,16*S*)-8,16-Bis(decyl)-6,9,12,15,18-Pentaoxa-25-Azatetracyclo [21.3.1.0^5,26^.0^19,24^]heptacosa-1,3,5(26),19,21,23-Hexaen-27-one [(*S*,*S*)-**1**]: See Scheme 2

Macrocycle (*S*,*S*)-**1** was prepared in the same way as described above for (*R*,*R*)-**1**, starting from 4,5-dihydroxyacridin-9(10*H*)-on (**5**) (1.20 g, 5.29 mmol) and enantiopure glycol derivative (*S*,*S*)-**10** (4.99 g, 6.37 mmol). Yield: 774 mg (22%). ${\left[\alpha \right]}_{D}^{25}$ = +23.08 (c = 0.43, CH_2_Cl_2_). The other physical and spectroscopic data of (*S*,*S*)-**1** concurred with those of (*R*,*R*)-**1**.

#### 4.2.5. (8*R*,16*R*)-8,16-Bis(decyl)-27-Phenyl-6,9,12,15,18-Pentaoxa-25-Azatetracyclo[21.3.1.0^5,26^.0^19,24^]heptacosa-1,3,5(26),19,21,23(27),24-Heptaene [(*R*,*R*)-2]: See Scheme 2

##### Procedure “**A**”

9-Phenylacridine-4,5-diol (**9**) was prepared according to the literature [31]. A mixture of dihydroxy derivative **9** (1.00 g, 3.48 mmol), didecyl-substituted tetraethylene glycol ditosylate (*R*,*R*)-**10** (3.27 g, 4.18 mmol), finely powdered anhydrous caesium carbonate (9.07 mg, 27.84 mmol) and dry and pure DMSO (200 mL) were stirred vigorously under an argon atmosphere at room temperature for 15 min, then the temperature of the mixture was raised to 60 °C and kept at this temperature for 1 week. Water (500 mL) was added to the reaction mixture and it was extracted with ethyl acetate (3 × 500 mL). The combined organic phase was extracted with water (6 × 500 mL) and then with saturated aqueous sodium chloride solution (2 × 500 mL) to remove most of the DMSO. The organic phase was dried over magnesium sulphate and filtered and the solvent was evaporated. The crude product was purified by column chromatography on silica gel, using dichloromethane as an eluent. The product needed further purification by PTLC on silica gel with dichloromethane as an eluent to gain (*R*,*R*)-**2** (126 mg, 5%) as light brown crystals.

M.p. > 360 °C. *R*_f_ = 0.42 (silica gel TLC, dichloromethane). ${\left[\alpha \right]}_{D}^{25}$ = −23.12 (c = 0.42, CH_2_Cl_2_). ^1^H-NMR (CDCl_3_): δ [ppm]: 0.89 (t, *J* = 6.7 Hz, 6H, CH_3_ groups located at the end of the decyl chains); 1.20–1.75 (m, 36H, CH_2_ groups of the decyl chains); 3.75–3.94 (m, 6H, two CH_2_ groups of the macrocyclic ring and the two CH groups of the macroring attached to the decyl chains); 4.00–4.18 (m, 4H, CH_2_ groups of the macroring); 4.33–4.47 (m, 4H, CH_2_ groups of the macroring near the aromatic moiety); 6.91 (d, *J* = 7.5 Hz, 2H, ArCH groups of the heterocyclic moiety near the macrocyclic ring); 7.19 (d, *J* = 8.8 Hz, 2H, ArCH groups of the heterocyclic moiety); 7.29 (t, *J* = 8.3 Hz, 2H, ArCH groups of the heterocyclic moiety); 7.42 (d, *J* = 7.0 Hz, 2H, aromatic protons at the ortho positions of the phenyl group attached to the acridine unit); 7.51–7.61 (m, 3H, the other protons of the phenyl group attached to the acridine unit). ^13^C-NMR (CDCl_3_): δ [ppm]: 14.29 (CH_3_ groups located at the end of the decyl chains); 22.86 (CH_2_ of decyl chains); 25.84 (CH_2_ of decyl chains); 29.12 (CH_2_ of decyl chains); 29.51 (CH_2_ of decyl chains); 29.81 (CH_2_ of decyl chains); 29.91 (CH_2_ of decyl chains); 30.38 (CH_2_ of decyl chains); 32.09 (CH_2_ of decyl chains); 32.29 (CH_2_ of decyl chains); 70.73 (CH_2_ groups of the macroring); 71.55 (CH_2_ groups of the macroring); 73.28 (CH_2_ groups of the macroring); 78.37 (CH groups of the macroring attached to the decyl chains); 106.75 (ArCH groups of the heterocyclic moiety); 118.43 (ArCH groups of the heterocyclic moiety); 125.90 (ArC); 126.73 (ArCH); 128.29 (ArCH); 128.51 (ArCH); 130.54 (ArCH); 136.91 (ArC); 141.15 (ArC); 146.26 (ArC); 154.86 (ArC). IR: ν_max_ [cm^−1^]: 3363, 3062, 2957, 2921, 2851, 1731, 1650, 1632, 1567, 1469, 1462, 1416, 1262, 1232, 1107, 1088, 1023, 802, 756, 728, 703, 671, 665. HRMS: *m*/*z* = [MH^+^]: 726.50855, [M]: 725.50192. Anal. Calcd. for C_47_H_67_NO_5_: C 77.75; H 9.30; N 1.93. Found: C 77.71; H 9.29; N 1.92. Enantiomeric purity was determined by chiral HPLC and found to be >98%.

##### Procedure “**B**”

The crown compound (*R*,*R*)-**1** (1.70 g, 2.55 mmol) and phosphorus pentachloride (0.53 g, 2.55 mmol) were dissolved in phosphoryl chloride (10 mL, 109.60 mmol) and the temperature of the resulting mixture was raised to 90 °C and stirred at this temperature for 2 h under an argon atmosphere. The reaction mixture was slowly poured into a mixture of trimethylamine (400 mL of aqueous solution, 40 m/m%) and ice (400 g) with external cooling (ice water bath). (A large excess of the trimethylamine solution was required because of the acid-sensitivity of the chloro-compound (*R*,*R*)-**11**.) After destroying the excess reagent, the pH of the solution was adjusted to 9 with aqueous hydrochloric acid (10 m/m%) and extracted with diethyl ether (4 × 500 mL). The combined organic phase was dried over magnesium sulphate and filtered and the solvent was removed. The yellow crystalline crude product (*R*,*R*)-**11** was obtained quantitatively and was used without purification and characterization because of its degradability.

A solution of crude (*R*,*R*)-**11** (1.73 g, 2.53 mmol) in dry and pure toluene (30 mL) was added dropwise to a stirred suspension of phenylmagnesium bromide (3 M in THF, 4.22 mL, 12.65 mmol), palladium acetate (29 mg, 0.13 mmol), dilithium tetrachloro cuprate (0.1 M in THF, 1.30 mL, 0.13 mmol) and dry toluene (10 mL) under an argon atmosphere at room temperature. The resulting mixture was stirred for 24 h then cooled down to 0 °C. The solvent was removed and the residue was taken up in ethyl acetate (100 mL) and water (100 mL). The pH of the aqueous phase was adjusted to 7 using aqueous hydrochloric acid (10 m/m%). The phases were shaken well and separated. The aqueous phase was extracted with ethyl acetate (4 × 100 mL). The combined organic phase was dried over magnesium sulphate and filtered and the solvent was removed. The crude product was purified by column chromatography on silica gel using dichloromethane as an eluent, yielding (*R*,*R*)-**2** (220 mg, 12% (based on starting material (*R*,*R*)-**1**)), which was identical in every aspect to that prepared by Procedure “**A**”.

#### 4.2.6. (8S,16S)-8,16-Bis(decyl)-27-Phenyl-6,9,12,15,18-Pentaoxa-25-Azatetracyclo[21.3.1.0^5,26^.0^19,24^]heptacosa-1,3,5(26),19,21,23(27),24-Heptaene [(S,S)-2]: See Scheme 2

##### Procedure “**A**”

Macrocycle (*S*,*S*)-**2** was prepared in the same way as described above for (*R*,*R*)-**2**, starting from 9-phenylacridine-4,5-diol (**9**) (1.00 g, 3.48 mmol) and didecyl-substituted tetraethylene glycol ditosylate (*S*,*S*)-**10** (3.27 g, 4.18 mmol). Yield: 151 mg (6%). ${\left[\alpha \right]}_{D}^{25}$ = +21.13 (c = 0.40, CH_2_Cl_2_). The other physical and spectroscopic data of (*S*,*S*)-**2** concurred with those of (*R*,*R*)-**2**.

###### Procedure “**B**”

Macrocycle (*S*,*S*)-**2** was prepared in the same way as described above for (*R*,*R*)-**2**, starting from crown compound (*S*,*S*)-**1** (1.70 g, 2.55 mmol). Yield: 257 mg (14%) (based on the crown compound (*S*,*S*)-**1**). The other physical and spectroscopic data of (*S*,*S*)-**2** concurred with those of (*R*,*R*)-**2**.

### 4.3. Transport Studies

For the preparation of the source phases, amines were added to a molar equivalent amount of acetic acid, and the salts formed were dissolved in water (1 mol/L) unless otherwise indicated. The transported quantities were determined both by weight and UV absorbance measurements. For the measurements, the receiving phases were evaporated and the masses of the solid residues were determined by a Mettler Toledo XS105 microanalytical balance (Mettler Toledo, Columbus, OH, USA) to the nearest 0.1 mg. For UV-Vis measurements, the ammonium salts were dissolved uniformly in ethanol. Spectrophotometric determinations were carried out using a 5-point calibration curve as a function of the ammonium salt concentration in ethanol. UV-absorption spectra and calibration curves of amine derivatives are available in Supplementary Materials from Figure S17 to Figure S38. The error of the transported amounts of ammonium salts was smaller than ±1.0 mg based on both the weight and spectrophotometric measurements. In the case of the determination of the enantiomeric excess of chiral amine derivatives, the ammonium salts were liberated by adding 2 mL of 10 m/m% aqueous sodium hydroxide solution; these aqueous phases were extracted with dichloromethane (3 × 2 mL), dried over magnesium sulphate, filtered, evaporated and dissolved in dichloromethane. The resulting solutions were investigated by polarimetry to determine the ratios of the mixtures of stereoisomers. The optical purity data were determined by measuring the specific rotations of the samples (α) and comparing them to the optical rotation of the enantiomerically pure component (α_max_). The errors of the optical purity values were less than ±2.0%. The degree of membrane leakage was less than 5.0% for NEA (**21**), less than 2.0% for other aralkylammonium salts, and negligible for aliphatic amine derivatives. Unless otherwise noted, the measurements were carried out at 15 °C to avoid theevaporation of some solvents of the phases and to maintain constant volume ratios. During the transport time, the membrane phase was stirred at 200 rpm using a magnetic stirrer. In order to regenerate the membrane, the bulk (dichloromethane) phase was separated from the source phase with a shaking funnel, the organic phase was then extracted with 3 × 2 mL of aqueous 10 m/m% acetic acid solution to remove the amines. The organic phase was dried over magnesium sulphate, filtered and evaporated. The purity of the resulting carrier was checked by TLC. All the values reported are derived from the average of three measurements.

### 4.4. Spectrophotometric Investigations

The concentrations of the solutions of macrocycles were 50 μmol/L in acetonitrile for the UV-Vis measurements and 5 μmol/L in acetonitrile for the fluorescence titrations. For spectrophotometric titrations, the hydrogen perchlorate salts of the primary amines in acetonitrile were added to the solutions containing the crown ether using a Hamilton syringe. The results were corrected with the background signal and dilution effect of the added ammonium salts.

The stability constants of the complexes were determined by a global nonlinear regression analysis using OriginPro 8.6 (OriginLab Corp., Northampton, MA, USA) software. For the determination of the complex stability constant based on the observed partial fluorescence quenching by complexation, the following equation was used [46]:

(2) $$\frac{F_{0}-F}{F_{0}-F_{c}}=\frac{\left[H\right]+\left[L\right]+\frac{1}{K_{a}}-\sqrt{{\left(\left[H\right]+\left[L\right]+\frac{1}{K_{a}}\right)}^{2}-4\cdot \left[H\right]\cdot \left[L\right]}\;}{2\cdot \left[H\right]},$$

where *F* is the measured fluorescence intensity, *F_0_* is the starting fluorescence of the host molecule, *F_c_* is the fluorescence of the fully complexed host, *K_α_* is the association constant of the complex, (*H*) is the concentration of the host and (*L*) is the concentration of the added ligand at a given point of titration. For easier handling, the equation has been aligned to *F*:

(3) $$F=F_{0}-\left(F_{0}-F_{c}\right)\cdot \frac{\left[H\right]+\left[L\right]+\frac{1}{K_{a}}-\sqrt{{\left(\left[H\right]+\left[L\right]+\frac{1}{K_{a}}\right)}^{2}-4\cdot \left[H\right]\cdot \left[L\right]}}{2\cdot \left[H\right]}.$$

*F_0_* is a wavelength-dependent variable. During fitting, *F_c_* and *K_α_* were defined as parameters, while the values of (*H*) and (*L*) were given as constants.

Fluorescence titration curves and fitted nonlinear functions of enantiopure macrocycles for determining the complex stability constants toward enantiomers of various chiral protonated amine derivatives are available in Supplementary Materials from Figure S39 to Figure S47.

### 4.5. Electrochemical Measurements

For potentiometric measurements, a Philips IS-561 (Glasblaserei Moller, Switzerland) electrode body was used with an Ag/AgCl/3 mol/L KCl//1 mol/L KCl double-junction reference electrode (Metrohm, Herisau, Switzerland) in a Radelkis OP-208/1 precision pH-meter (Radelkis Ltd., Budapest, Hungary). Amounts of 10^−3^ mol/L of aqueous racemic solutions of the investigated protonated primary amines were used as the inner filling solution during calibrations, even in the cases of selectivity measurements.

To incorporate ligands **1** and **2** into the potentiometric sensor, the following membrane composition was made: 1 mg of ionophore, 33 mg of PVC powder (Corvic S 704, ICI), 66 mg of dioctyl sebacate (DOS) apolar plasticizer, and one equivalent (regarding the ionophore) potassium tetrakis(4-chlorophenyl)borate lipophilic ionic additive were dissolved in 2 mL of THF. This solution was placed into a 20 mm diameter teflon ring. After the evaporation of the solvent, a 7 mm diameter disk was cut out from the membrane and incorporated into the electrode body.

All the potentiometric measurements were carried out in distilled water at room temperature. All the data reported are the average of at least three independent measurements. For the measurements, the sample solutions were stirred at 100 rpm using a magnetic stirrer. The EMF of the cell was measured by varying the concentration of the stirred solutions in the range from 1.0 × 10^−7^ to 1.0 × 10^−1^ M by serial dilution. For calibration, the EMF values were recorded both with increasing and decreasing concentrations. Each point of the diagrams is the average of three independent measurements. The deviation was below ±1.0 mV in every case. Between two different sample solutions, the electrode pair was washed with distilled water and then wiped off. The response time of the membrane electrodes—i.e., the time in which the stable and constant potentials were recorded—was determined by measuring the potentials at different times. The potential values were recorded 60 s after immersing the electrode into the test solution. The lower detection limit was determined by a graphical method according to the definition reported in the literature [47]. The potentiometric selectivity coefficients were determined by the separate solution method based on the Nikolsky–Eisenman equation [47] from the EMF data measured for the hydrochloric acid salts of the enantiomers of amines. The activity coefficients in the electrochemical relationships used in the calculations were estimated by the concentration data and were not corrected. To quantify the membrane selectivity, the potentiometric selectivity coefficients were calculated by Equation (4):

(4) $$\log K_{\left(R/S\right)}^{\mathrm{pot}}=\frac{{\mathrm{EMF}}_{R}-{\mathrm{EMF}}_{S}}{S}+\log\frac{a_{R}}{a_{S}{}^{\left(z_{R}/z_{S}\right)}},$$

where $\log K_{\left(R/S\right)}^{\mathrm{pot}}$ depends on the membrane composition, the complex stability constants of the ionophore with the individual ions and the lipophilicity of the ions measured. In our case, a*_R_* and a*_S_* are the activities of the enantiomers in each solution and induce EMF*_R_* and EMF*_S_* potentials respectively, *S* is the slope of the potentiometric calibration curve as a function of ammonium ion activity in the solution and z*_R_* and z*_S_* are the charges of the enantiomers.

Electrochemical calibration curves and potentiometric selectivity measurements of ion-selective membrane electrode are available in Supplementary Materials from Figure S48 to Figure S53.

## Figures and Tables

**Table 1:** Effect of counter ions on the transport of racemic protonated NEA (**21**) after 10 h of transport time using macrocycle (*R*,*R*)-**2** as a carrier.

Counter Ions	Transported Amount (%)	Optical Purity (%)
acetate	45	5
*t*-butyl sulfonate	42	7
chloride	42	7
*p*-toluenesulfonate	38	5

**Table 2:** The effect of temperature on the transport of racemic acetic acid salt of NEA (**21**) after 2 h of transport time using macrocycle (*R*,*R*)-**2** as a carrier.

Temperature (°C)	Transported Amount (%)	Optical Purity (%)
10	10	68
15	12	62
20	16	53

**Table 3:** Selectivity of ligands (*R*,*R*)-**1** and (*R*,*R*)-**2** for the enantiomers of aralkylamine hydrochloride salts **21**–**24** expressed as the difference in EMF values in 10^−3^ M aralkylammonium chloride solutions and the corresponding potentiometric and enantioselectivity factors obtained by carrying out 12 measurements.

Macrocycle	Ammonium Salts	ΔEMF ^1^ (mV)	Potentiometric Selectivity ^2^ $K_{\left(R/S\right)}^{pot}$	Enantioselectivity ^3^ $\mathrm{Enantioselectivity}\frac{\beta_{RL}^{w}}{\beta_{SL}^{w}}$	Estimated Δlog*K*_(*R*/*S*)_^4^
**1**	**21**	2.5	0.89 ± 0.02	1.12 ± 0.03	0.05 ± 0.01
**1**	**22**	≈0	≈1	≈1	≈0
**1**	**23**	≈0	≈1	≈1	≈0
**1**	**24**	≈0	≈1	≈1	≈0
**2**	**21**	13.9	0.55 ± 0.02	1.83 ± 0.07	0.26 ± 0.02
**2**	**22**	11.3	0.60 ± 0.03	1.66 ± 0.09	0.22 ± 0.02
**2**	**23**	−13.4	1.79 ± 0.10	0.56 ± 0.03	−0.25 ± 0.02
**2**	**24**	3.9	0.85 ± 0.03	1.18 ± 0.05	0.07 ± 0.02

^1^$∆\mathrm{EMF}\;=\bar{\;{\mathrm{EMF}}_{R}}-\bar{{\mathrm{EMF}}_{S}}$. ^2^$K_{\left(R/S\right)}^{\mathrm{pot}}\;\approx \;\frac{\beta_{SL}^{w}}{\beta_{RL}^{w}}$. ^3^$\beta_{SL}^{w}$: the stability constant for the complex of the (*R*,*R*)-host molecule with the (*S*)-guest molecule; $\beta_{RL}^{w}$: stability constant for the complex of the (*R*,*R*)-host molecule with the (*R*)-guest molecule. ^4^$∆\log K_{\left(R/S\right)}=\;\log K_{R}-\log K_{S}$, where logK is the logarithm of the complex stability constant of the (*R*,*R*)-host molecule with the given enantiomer of the guest molecule, in this case estimated by the logarithm of the potentiometric selectivity coefficients.

## Supplementary Materials

The following are available online. Figure S1. ^1^H-NMR spectrum of intermediates (*R*)-3 and (*S*)-3; Figure S2. ^13^C-NMR spectrum of intermediates (*R*)-3 and (*S*)-3; Figure S3. ^1^H-NMR spectrum of intermediate 5: Figure S4. ^13^C-NMR spectrum of intermediate 5; Figure S5. ^1^H-NMR spectrum of intermediate 9 (after shaking with D_2_O); Figure S6. ^13^C-NMR spectrum of intermediate 9; Figure S7. ^1^H-NMR spectrum of intermediates (*R*,*R*)-10 and (*S*,*S*)-10; Figure S8. ^13^C-NMR spectrum of intermediates (*R*,*R*)-10 and (*S*,*S*)-10; Figure S9. ^1^H-NMR spectrum of compounds (*R*)-4 and (*S*)-4; Figure S10. ^13^C-NMR spectrum of compounds (*R*)-4 and (*S*)-4, Figure S11. ^1^H-NMR spectrum of macrocycles (*R*,*R*)-1 and (*S*,*S*)-1; Figure S12. ^13^C-NMR spectrum of macrocycles (*R*,*R*)-1 and (*S*,*S*)-1, Figure S13. ^1^H-NMR spectrum of macrocycles (*R*,*R*)-2 and (*S*,*S*)-2; Figure S14. ^13^C-NMR spectrum of macrocycles (*R*,*R*)-2 and (*S*,*S*)-2; Figure S15. Chromatogram for macrocycles (*R*,*R*)-**1** and (*S*,*S*)-1; Figure S16. Chromatogram for macrocycles (*R*,*R*)-2 and (*S*,*S*)-2; Figure S17. UV-absorption spectrum of amine derivative 12; Figure S18. UV-absorption calibration curve for amine derivative 12 at the excitation wavelength of 202 nm; Figure S19. UV-absorption spectrum of amine derivative 13; Figure S20. UV-absorption calibration curve for amine derivative 13 at the excitation wavelength of 202 nm; Figure S21. UV-absorption spectrum of amine derivative 14; Figure S22. UV-absorption calibration curve for amine derivative 14 at the excitation wavelength of 202 nm; Figure S23. UV-absorption spectrum of amine derivative 15; Figure S24. UV-absorption calibration curve for amine derivative 15 at the excitation wavelength of 248 nm; Figure S25. UV-absorption spectrum of amine derivative 16; Figure S26. UV-absorption calibration curve for amine derivative 16 at the excitation wavelength of 207 nm; Figure S27. UV-absorption spectrum of amine derivative 17; Figure S28. UV-absorption calibration curve for amine derivative 17 at the excitation wavelength of 207 nm; Figure S29. UV-absorption spectrum of amine derivative 18; Figure S30. UV-absorption calibration curve for amine derivative 18 at the excitation wavelength of 226 nm; Figure S31. UV-absorption spectrum of amine derivative 19; Figure S32. UV-absorption calibration curve for amine derivative 19 at the excitation wavelength of 204 nm; Figure S33. UV-absorption spectrum of amine derivative 20; Figure S34. UV-absorption calibration curve for amine derivative 20 at the excitation wavelength of 280 nm; Figure S35. UV-absorption spectrum of amine derivative 21; Figure S36. UV-absorption calibration curve for amine derivative 21 at the excitation wavelength of 280 nm; Figure S37. UV-absorption spectrum of amine derivative 22; Figure S38. UV-absorption calibration curve for amine derivative 22 at the excitation wavelength of 207 nm; Figure S39. Fluorescence titration of macrocycle (*S*,*S*)-2 with (*R*)-NEA (21)*HClO_4_; Figure S40. Fluorescence titration of macrocycle (*S*,*S*)-2 with (*S*)-NEA (21)*HClO_4_; Figure S41. Fitted nonlinear functions for determining the stability constants of (*S*,*S*)-2 with the enantiomers of NEA (21)*HClO_4_; Figure S42. Fluorescence titration of macrocycle (*S*,*S*)-2 with (*R*)-PGME (23)*HClO_4_; Figure S43. Fluorescence titration of macrocycle (*S*,*S*)-2 with (*S*)-PGME (23)*HClO_4_; Figure S44. Fitted nonlinear functions for determining the stability constants of (*S*,*S*)-2 with the enantiomers of PGME (23)*HClO_4_; Figure S45. Fluorescence titration of macrocycle (*S*,*S*)-2 with (*R*)-PAME (24)*HClO_4_; Figure S46. Fluorescence titration of macrocycle (*S*,*S*)-2 with (*S*)-PAME (24)*HClO_4_; Figure S47. Fitted nonlinear functions for determining the stability constants of (*S*,*S*)-2 with the enantiomers of PAME (24)*HClO_4_; Figure S48. Calibration curve for racemic PEA (22)*HCl; Figure S49. Measurements to determine potentiometric selectivity toward enantiomers of PEA (22)*HCl; Figure S50. Calibration curve for racemic PGME (23)*HCl; Figure S51. Measurements to determine potentiometric selectivity toward enantiomers of PGME (23)*HCl; Figure S52. Calibration curve for racemic PAME (24)*HCl; Figure S53. Measurements to determine potentiometric selectivity toward enantiomers of PAME (24)*HCl.
