# Supplementary material for: Synthesis, Molecular Recognition Study and Liquid Membrane-Based Applications of Highly Lipophilic Enantiopure Acridino-Crown Ethers
Source: Molecules. 2020 May 31;25(11):2571. doi: 10.3390/molecules25112571 (PMC7321232; doi:10.3390/molecules25112571)
Supplement: Supplementary file 1 [file molecules-25-02571-s001.pdf]

SUPPORTING INFORMATION

Synthesis, molecular recognition study and liquid membrane-based applications of highly lipophilic enantiopure acridino-crown ethers

Ádám Golcs [1,*], Bálint Árpád Ádám [1], Viola Horváth [2], Tünde Tóth [1,3], Péter Huszthy [1]

[1] Department of Organic Chemistry and Technology, Budapest University of Technology and Economics, Szent Gellért tér 4., H-1111 Budapest, Hungary
[2] MTA-BME Computation Driven Chemistry Research Group, Szent Gellért tér 4., H-1521 Budapest, Hungary
[3] Institute for Energy Security and Environmental Safety, Centre for Energy Research, Konkoly-Thege Miklós út 29-33., H-1121 Budapest, Hungary
* Correspondence: golcs.adam@gmail.com

Contents

1. ^{1}H-NMR and ^{13}C-NMR spectra of the key intermediates used for preparation of new compounds (solvent: $CDCl_3$)
2. ^{1}H-NMR and ^{13}C-NMR spectra of the new compounds (solvent: $CDCl_3$)
3. Determination of enantiomeric purity of macrocycles by chiral HPLC
4. UV calibration curves for determining the transported amounts of various amine derivatives
5. Spectrophotometric determination of complex stability constants (log*K* values) of the reported enantiopure macrocycles (*R*,*R*)-**2** and (*S*,*S*)-**2** with the enantiomers of various chiral protonated amine derivatives
6. Electrochemical calibration curves and potentiometric selectivity measurements of ion-selective membrane electrode containing (*R*,*R*)-**2** as an ionophore

1. ^{1}H-NMR and ^{13}C-NMR spectra of the key intermediates used for preparation of new compounds (solvent: $CDCl_3$)

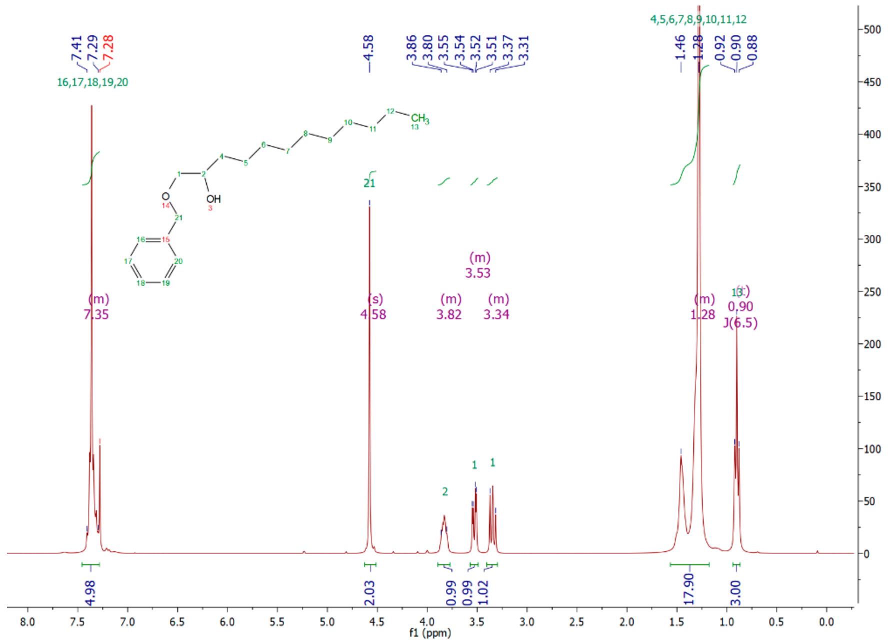

Figure S1. ^{1}H-NMR spectrum of intermediates (*R*)-**3** and (*S*)-**3**

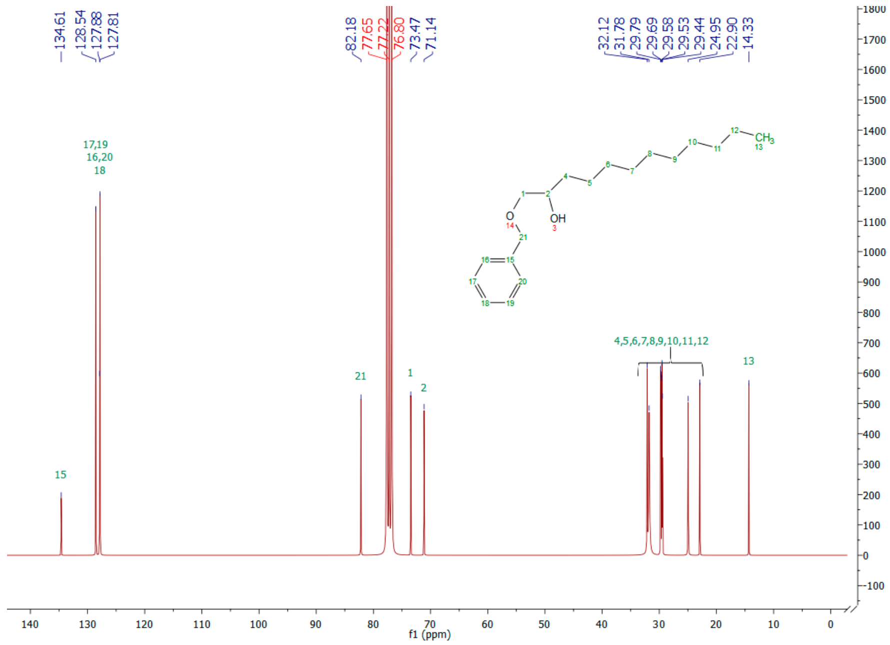

Figure S2. ^{13}C-NMR spectrum of intermediates (*R*)-**3** and (*S*)-**3**

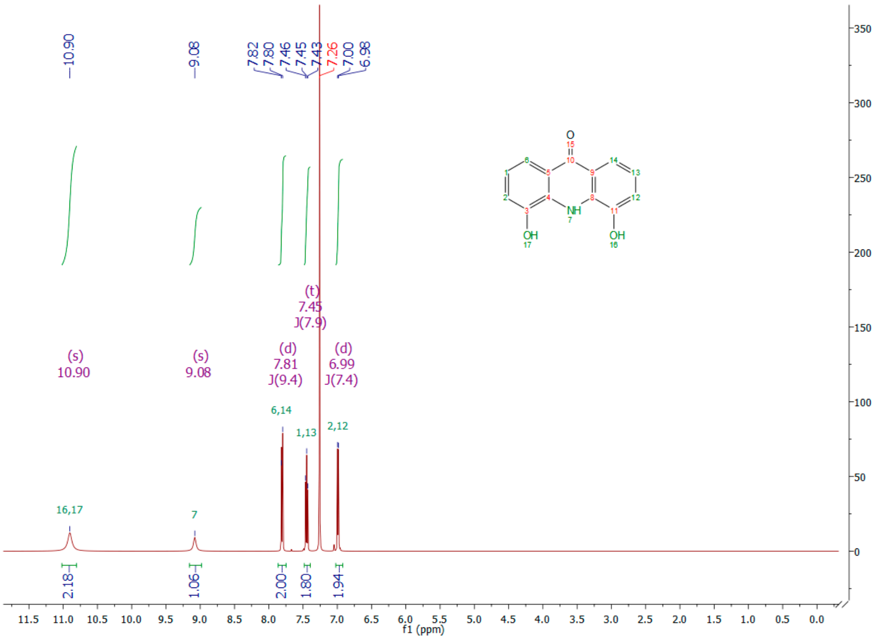

Figure S3. ^{1}H-NMR spectrum of intermediate **5**

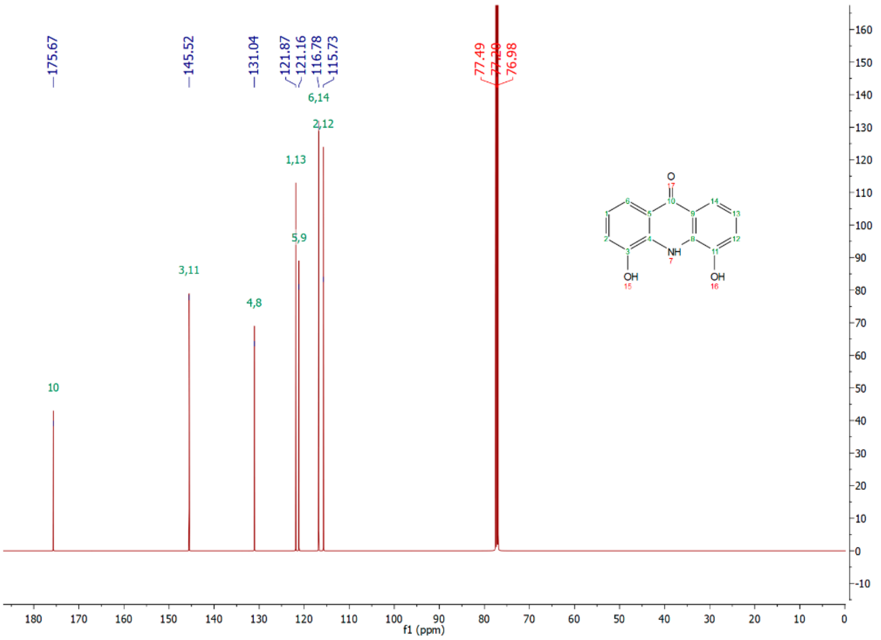

Figure S4. ^{13}C-NMR spectrum of intermediate **5**

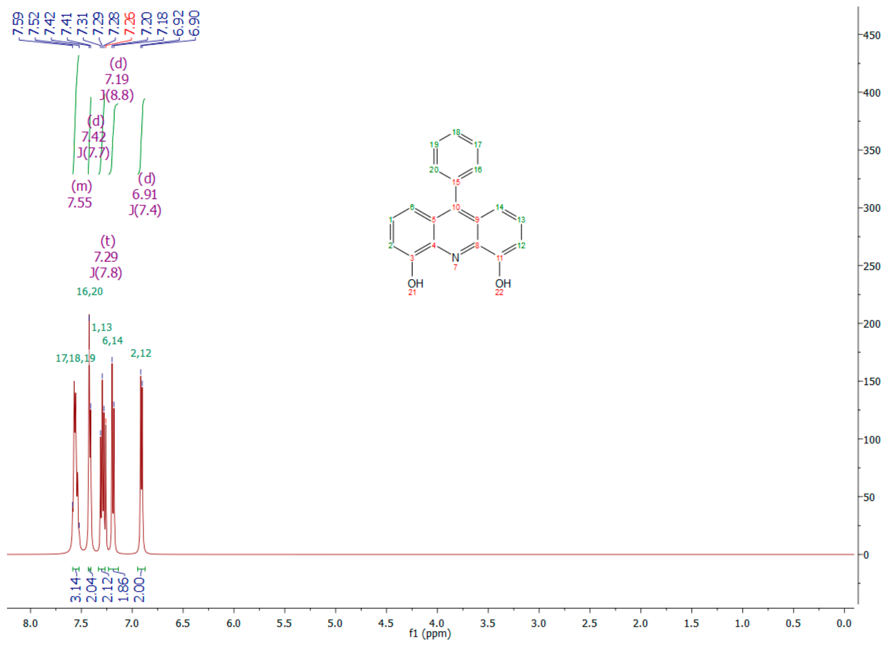

Figure S5. ^{1}H-NMR spectrum of intermediate **9** (after shaking with D_2O)

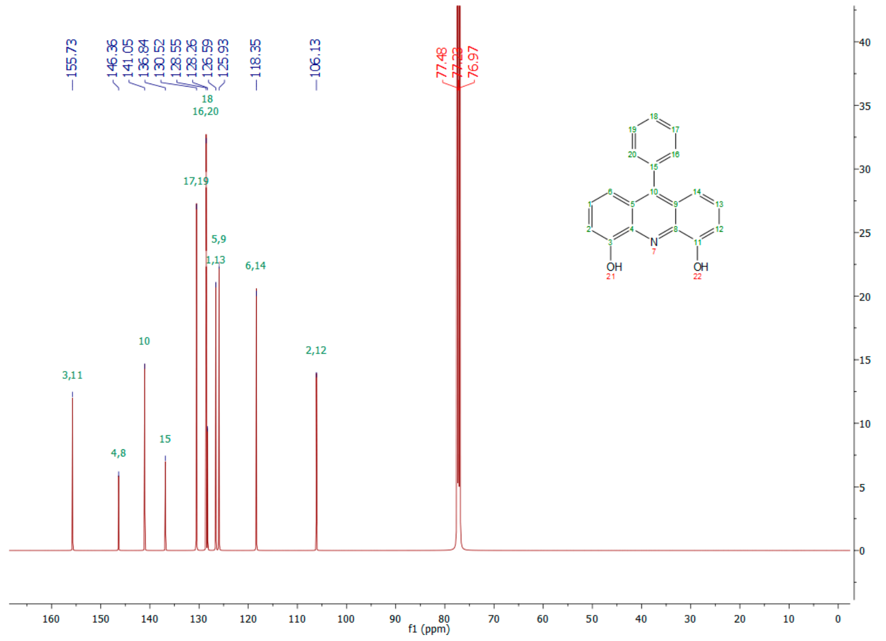

Figure S6. ^{13}C-NMR spectrum of intermediate **9**

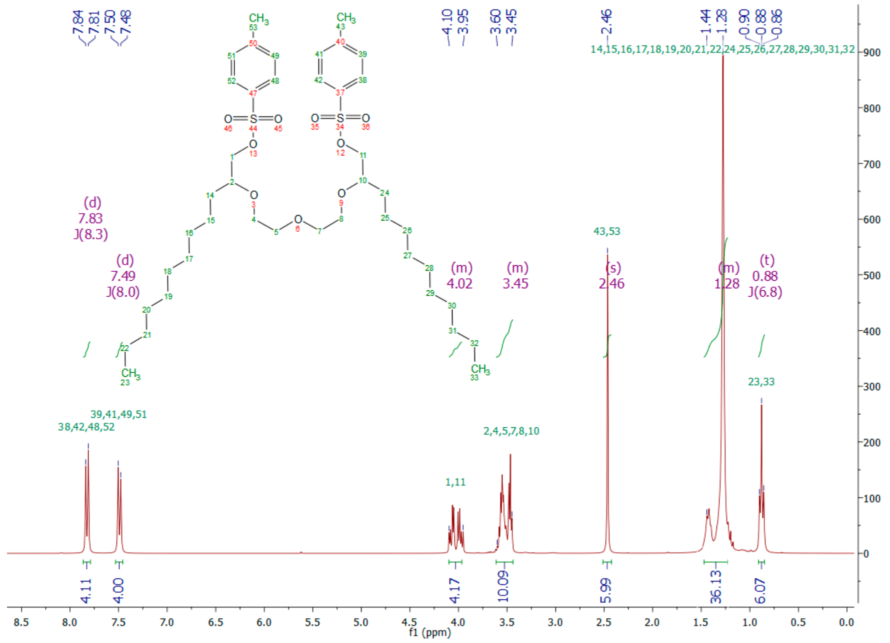

Figure S7. ^{1}H-NMR spectrum of intermediates (*R*,*R*)-**10** and (*S*,*S*)-**10**

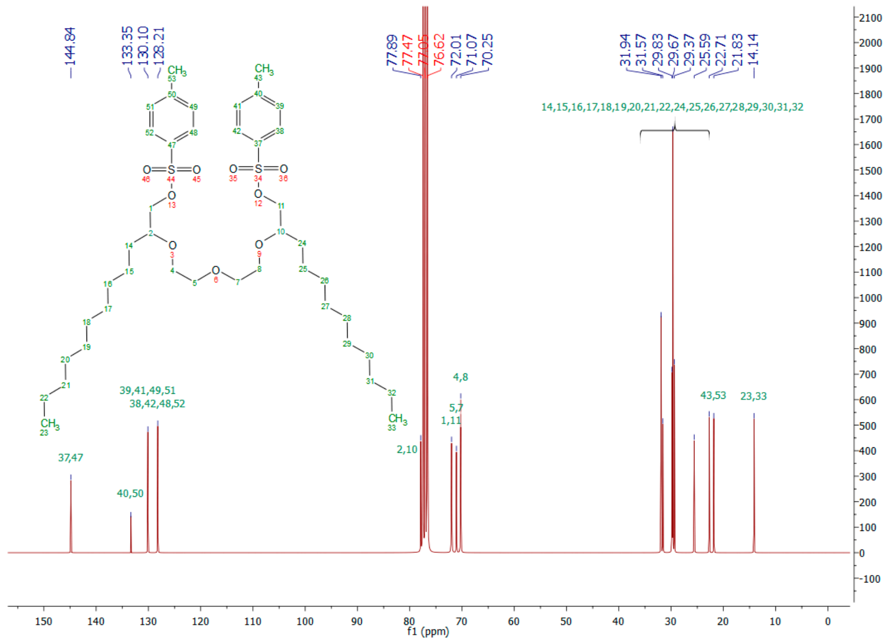

Figure S8. ^{13}C-NMR spectrum of intermediates (*R*,*R*)-**10** and (*S*,*S*)-**10**

2. ^{1}H-NMR and ^{13}C-NMR spectra of the new compounds (solvent: $CDCl_3$)

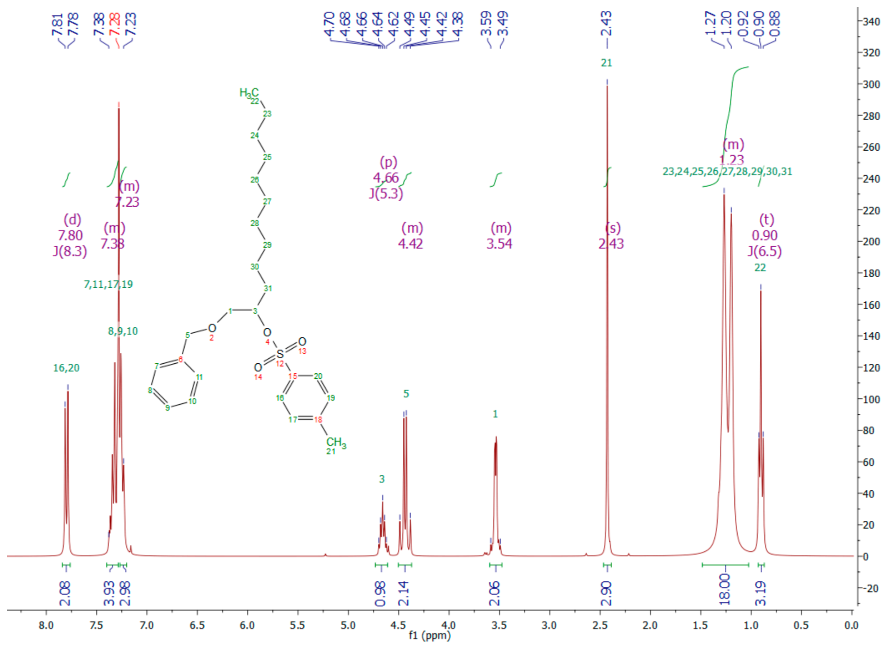

Figure S9. ^{1}H-NMR spectrum of compounds (*R*)-**4** and (*S*)-**4**

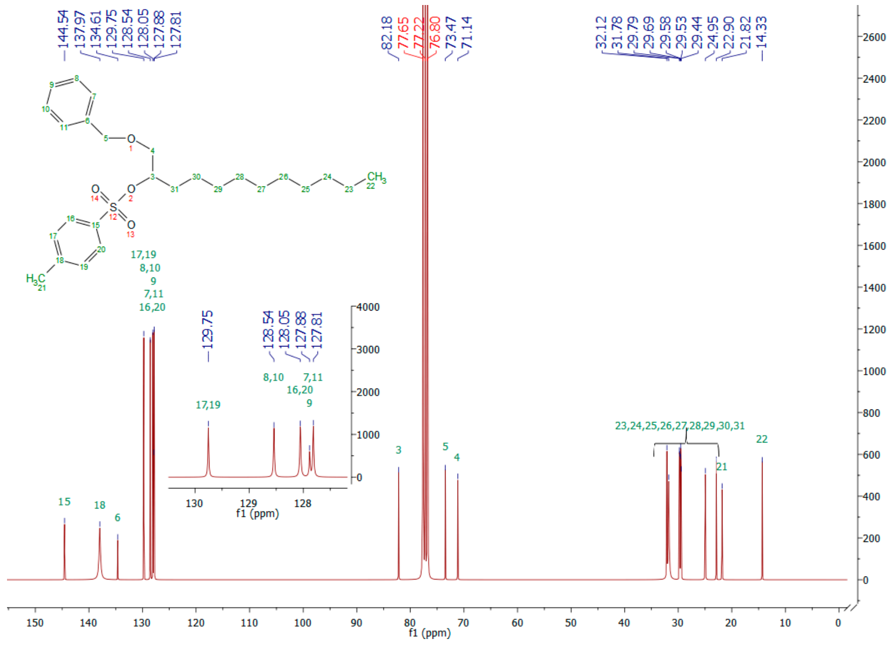

Figure S10. ^{13}C-NMR spectrum of compounds (*R*)-**4** and (*S*)-**4**

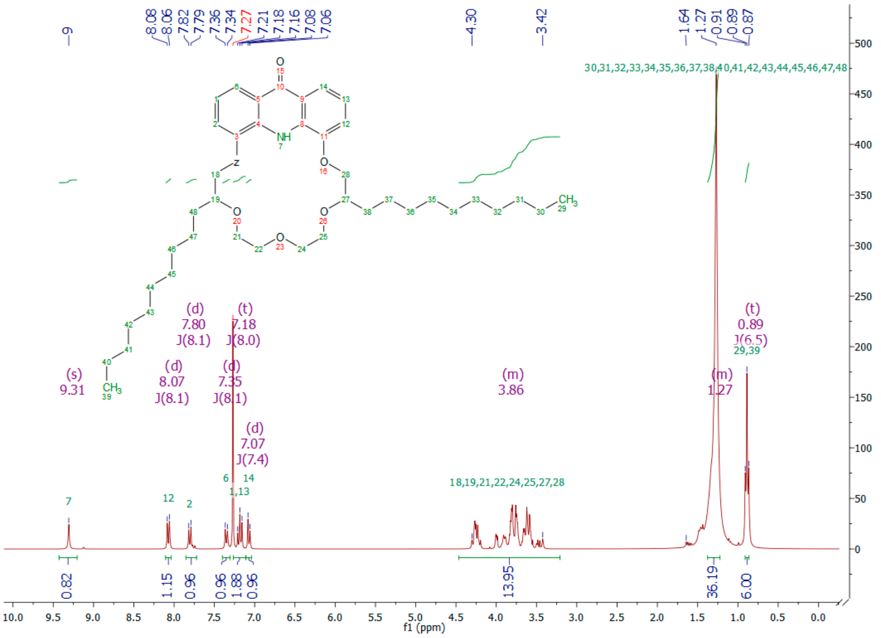

Figure S11. ^{1}H-NMR spectrum of macrocycles (*R*,*R*)-**1** and (*S*,*S*)-**1**

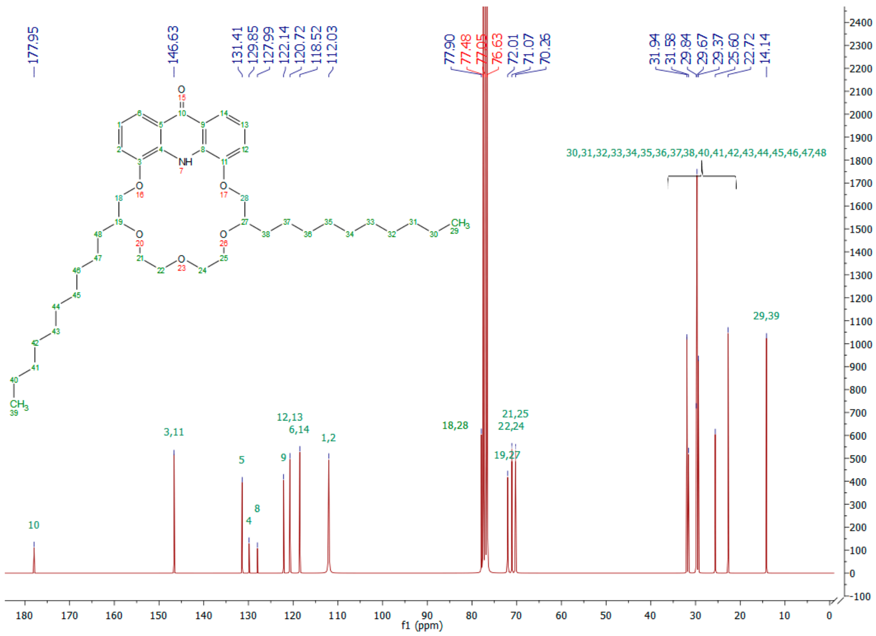

Figure S12. ^{13}C-NMR spectrum of macrocycles (*R*,*R*)-**1** and (*S*,*S*)-**1**

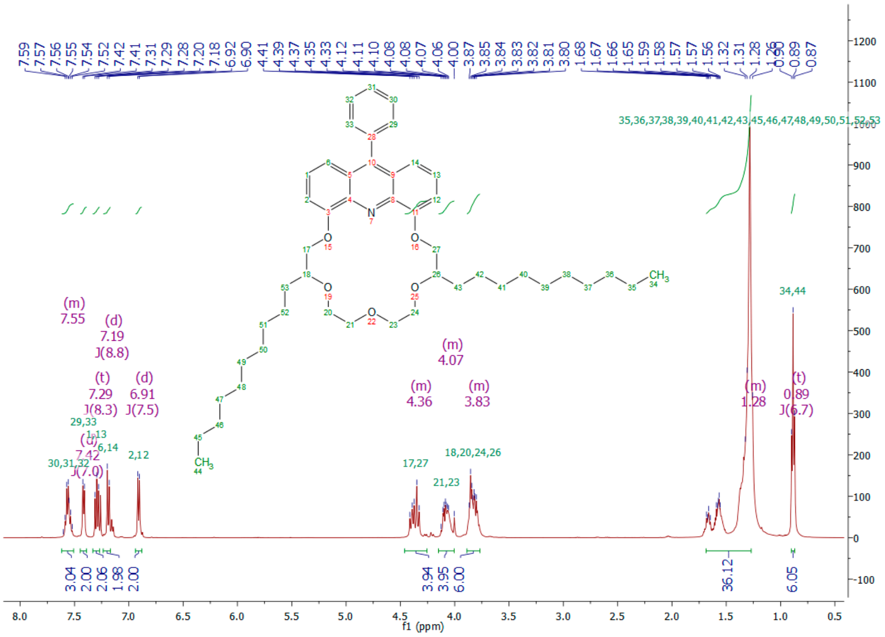

Figure S13. ^{1}H-NMR spectrum of macrocycles (*R*,*R*)-**2** and (*S*,*S*)-**2**

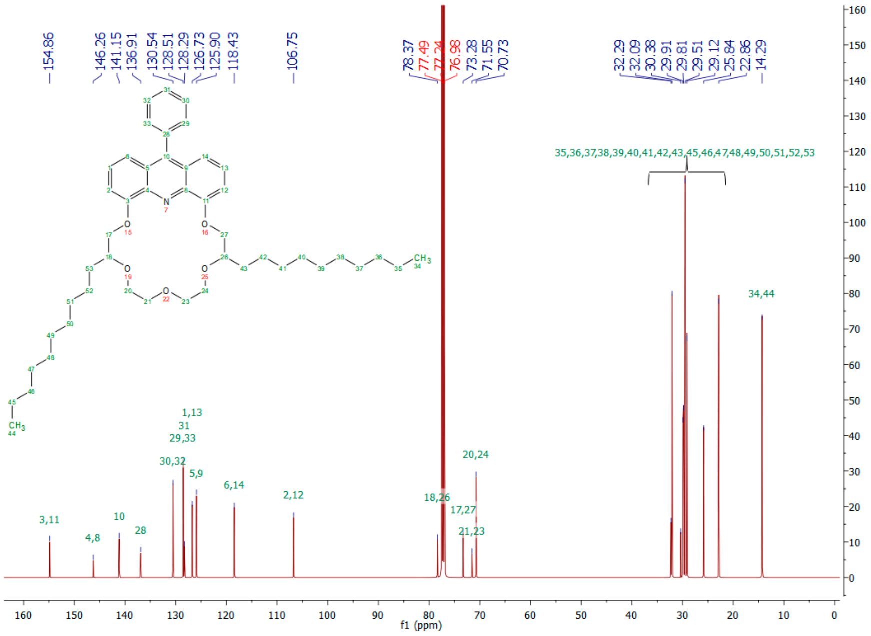

Figure S14. ^{13}C-NMR spectrum of macrocycles (*R*,*R*)-**2** and (*S*,*S*)-**2**

3. Determination of enantiomeric purity of macrocycles by chiral HPLC

Chiral chromatography was performed on a VWR Hitachi Elite (Hitachi Ltd., Japan) HPLC system, involving a Diode Array Detector L-2450, a LaChrom L-2310 pump, an L-2200 autosampler, and an L-2300 column oven, using a Reprosil Chiral MIA HPLC column (100 x 4,6 mm). Isocratic elution was applied with solvent system of 1:9 mixture of isopropyl alcohol/CH_3CN + 0.1 % trifluoroacetic acid and applying a flow rate of 0.4 mL/min at 20 °C. UV absorbance detection was carried out at 280 nm.

In case of crown ethers (*R*,*R*)-**1** and (*S*,*S*)-**1** a mixture containing both of the enantiomers with an enantiomeric ratio of (*R*,*R*):(*S*,*S*) = 1:2 was measured. Retention times: 9.14 min for (*R*)-enantiomer and 7.96 min for (*S*)-enantiomer. Enantiomeric purity was calculated from the ratios of the areas of chromatographic peaks and found to be > 97 %.

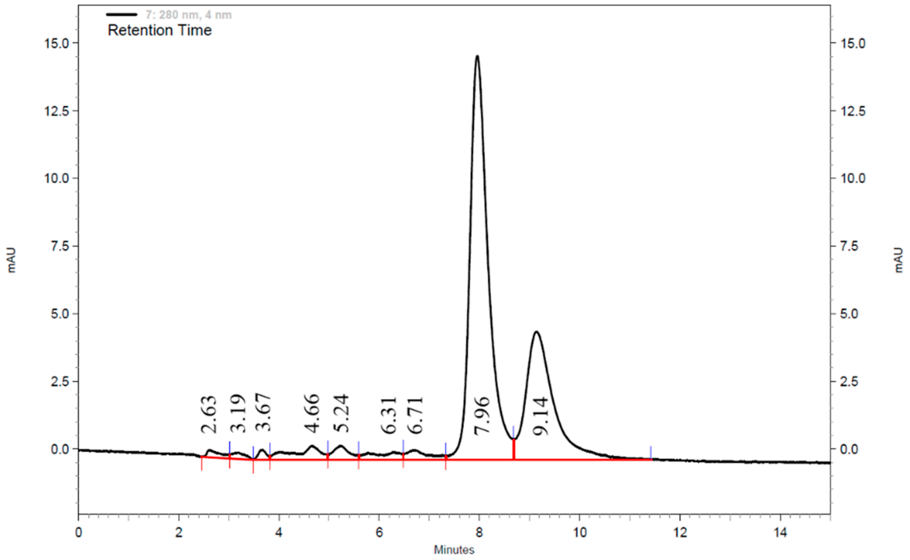

Peak	Retention Time	Area Percent	Capacity factor	Resolution (USP)	Asymmetry
1	2.63	0.82	0.53	0.00	0.00
2	3.19	0.69	0.86	0.00	0.00
3	3.67	0.66	1.14	0.00	0.00
4	4.66	3.06	1.71	1.86	0.00
5	5.24	1.86	2.05	0.85	0.00
6	6.31	1.81	2.68	1.44	0.00
7	6.71	1.80	2.91	0.48	0.00
8	7.96	60.09	3.64	1.73	0.00
9	9.14	29.21	4.32	1.50	0.00
Totals		100.00			

Figure S15. Chromatogram for macrocycles (*R*,*R*)-**1** and (*S*,*S*)-**1**

In case of crown ethers (*R*,*R*)-**2** and (*S*,*S*)-**2** a mixture containing both of the enantiomers with an enantiomeric ratio of (*R*,*R*):(*S*,*S*) = 1:2 was measured. Retention times: 9.62 min for (*R*)-enantiomer and 8.43 min for (*S*)-enantiomer. Enantiomeric purity was calculated from the ratios of the areas of chromatographic peaks and found to be > 98 %.

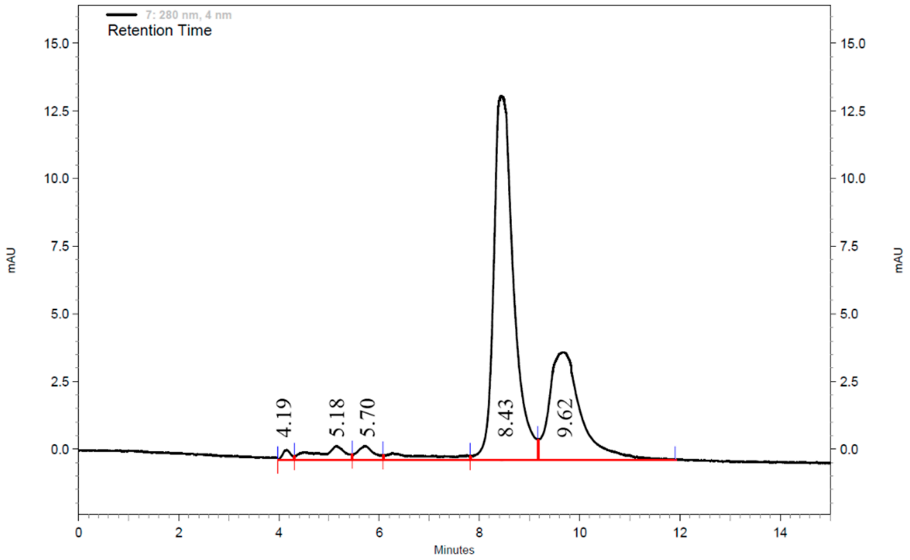

Peak	Retention Time	Area Percent	Capacity factor	Resolution (USP)	Asymmetry
1	4.19	1.58	1.44	0.00	0.00
2	5.18	5.23	2.01	1.76	0.00
3	5.70	3.59	2.31	0.92	0.00
4	8.43	60.05	3.90	1.71	0.00
5	9.62	29.55	4.59	1.60	0.00
Totals		100.00			

Figure S16. Chromatogram for macrocycles (*R*,*R*)-**2** and (*S*,*S*)-**2**

4. UV calibration curves for determining the transported amounts of various amine derivatives

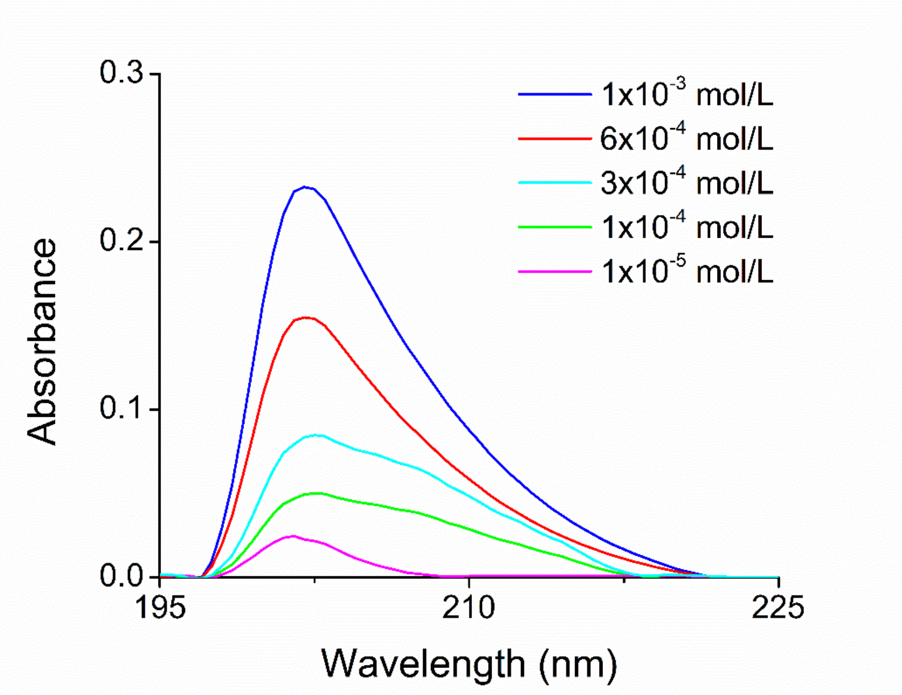

Figure S17. UV-absorption spectrum of amine derivative **12**

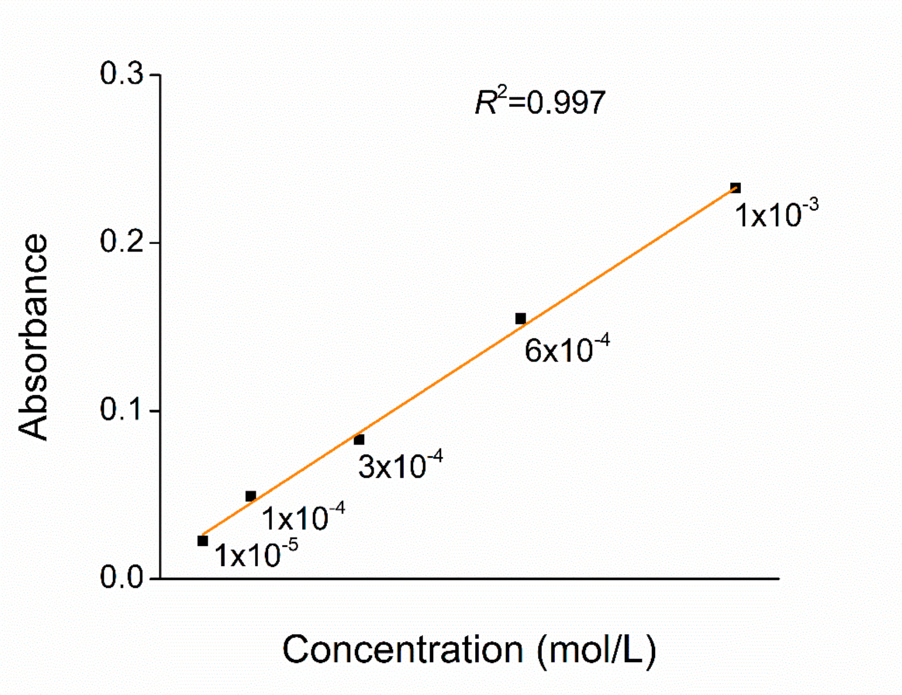

Figure S18. UV-absorption calibration curve for amine derivative **12** at the excitation wavelength of 202 nm

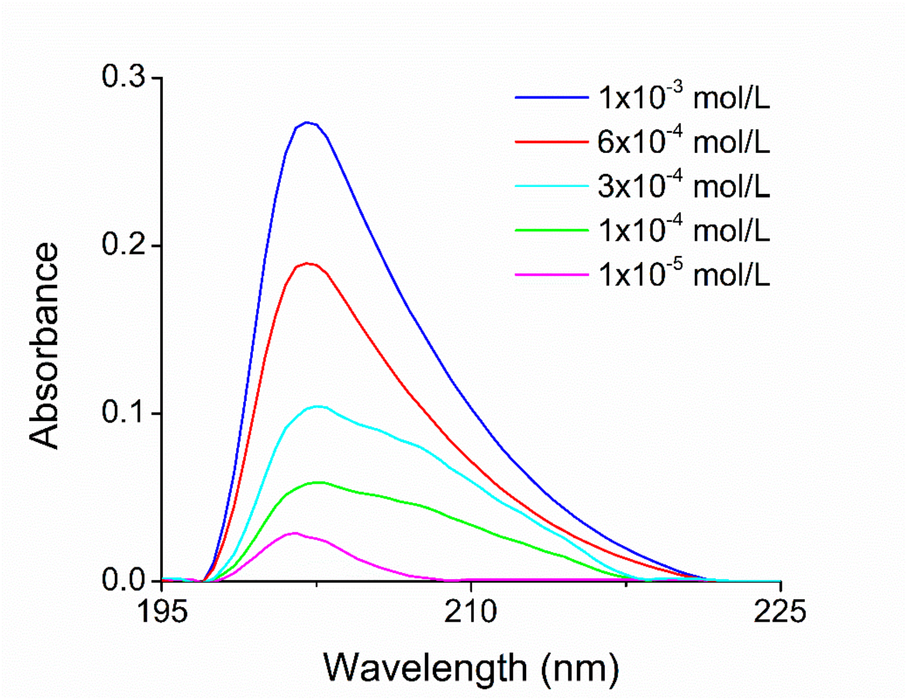

Figure S19. UV-absorption spectrum of amine derivative **13**

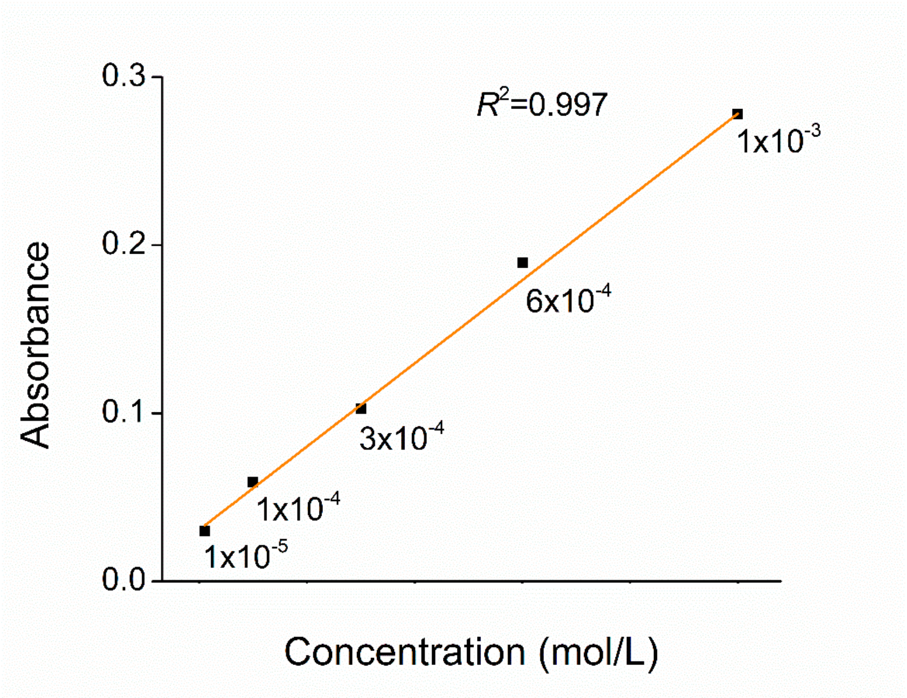

Figure S20. UV-absorption calibration curve for amine derivative **13** at the excitation wavelength of 202 nm

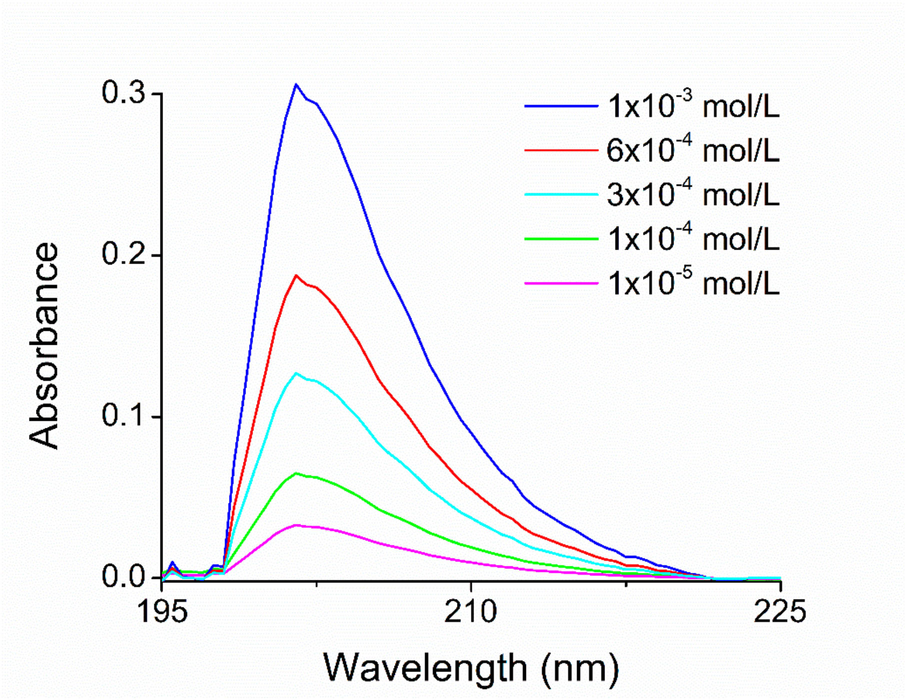

Figure S21. UV-absorption spectrum of amine derivative **14**

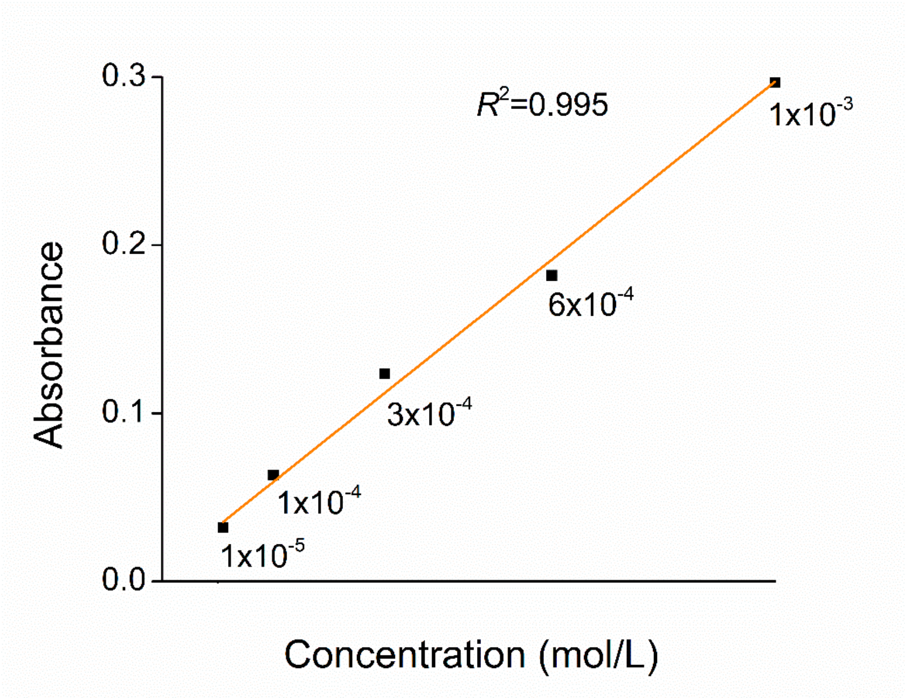

Figure S22. UV-absorption calibration curve for amine derivative **14** at the excitation wavelength of 202 nm

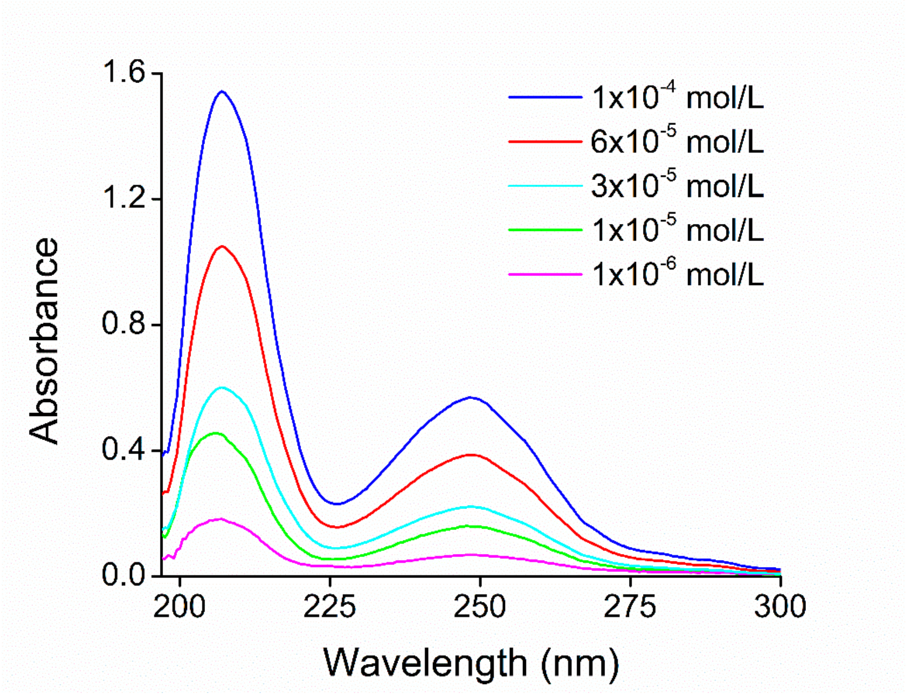

Figure S23. UV-absorption spectrum of amine derivative **15**

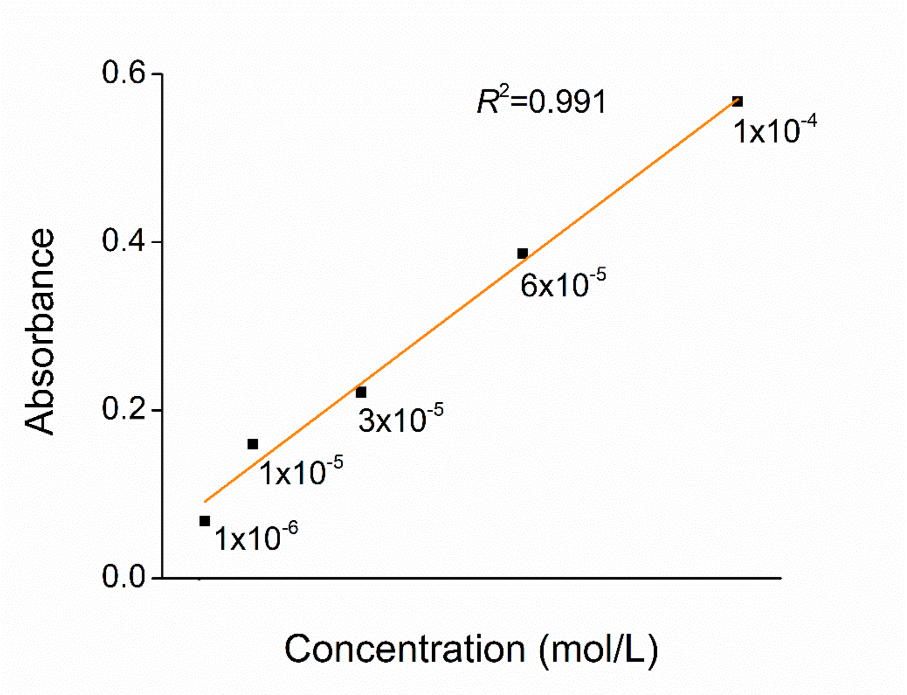

Figure S24. UV-absorption calibration curve for amine derivative **15** at the excitation wavelength of 248 nm

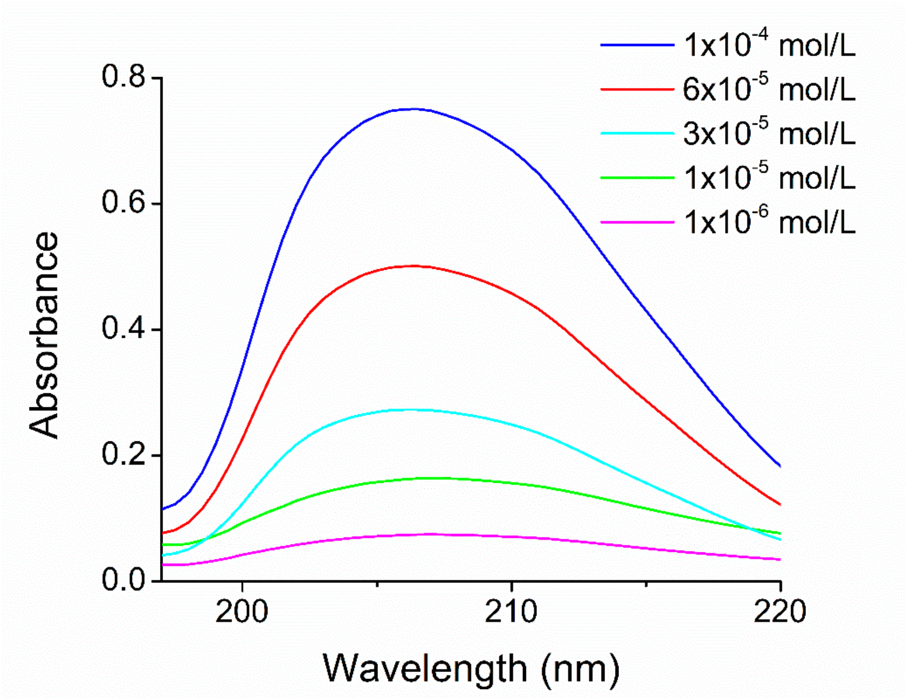

Figure S25. UV-absorption spectrum of amine derivative **16**

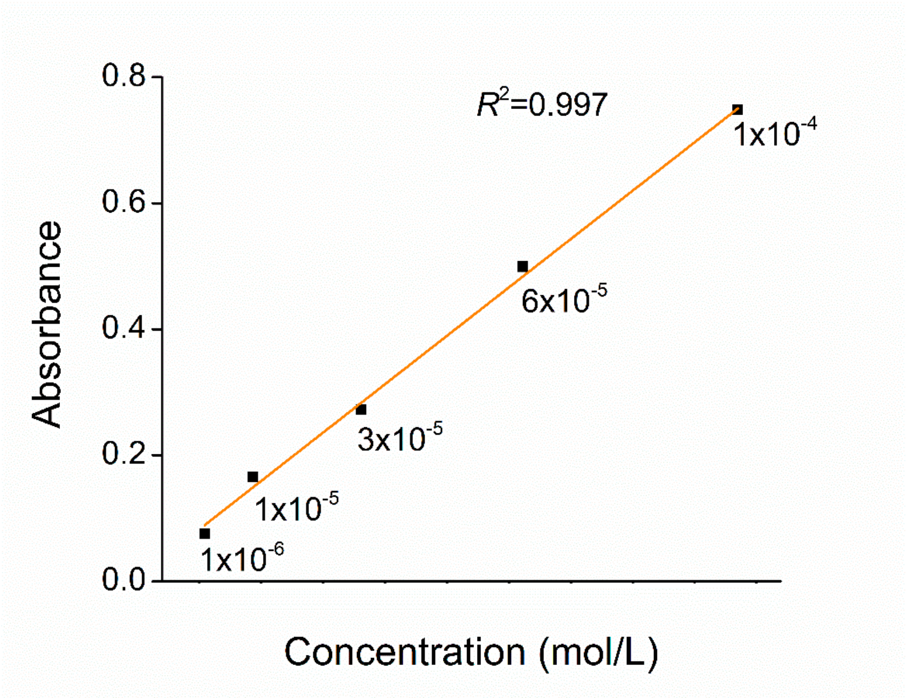

Figure S26. UV-absorption calibration curve for amine derivative **16** at the excitation wavelength of 207 nm

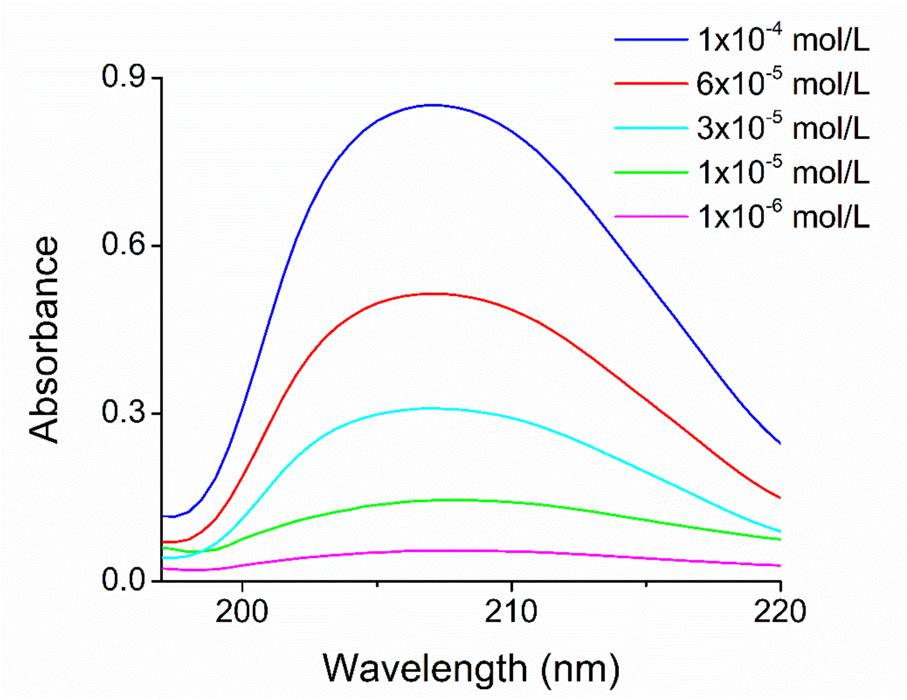

Figure S27. UV-absorption spectrum of amine derivative **17**

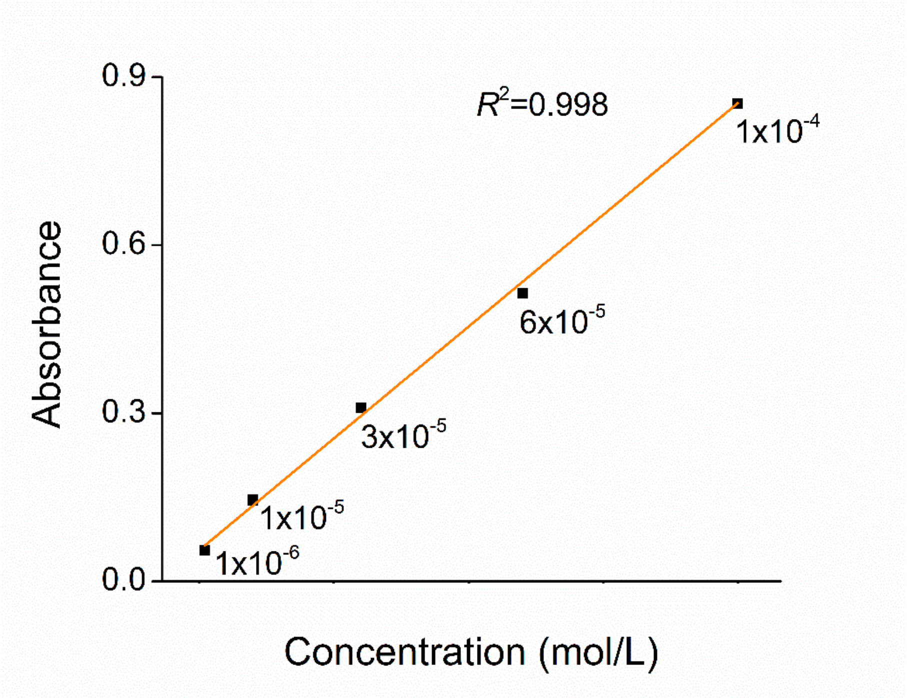

Figure S28. UV-absorption calibration curve for amine derivative **17** at the excitation wavelength of 207 nm

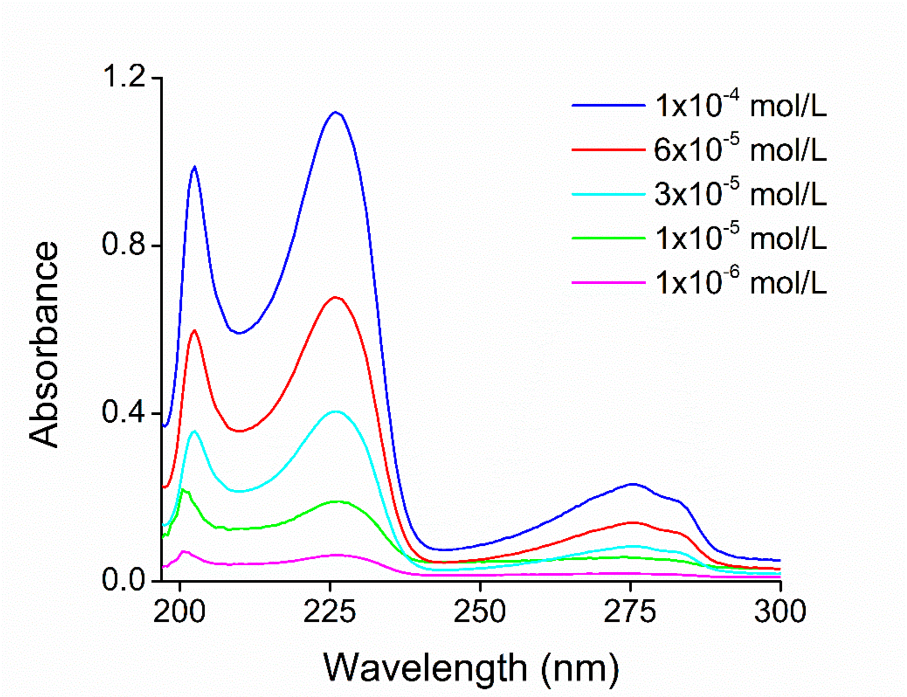

Figure S29. UV-absorption spectrum of amine derivative **18**

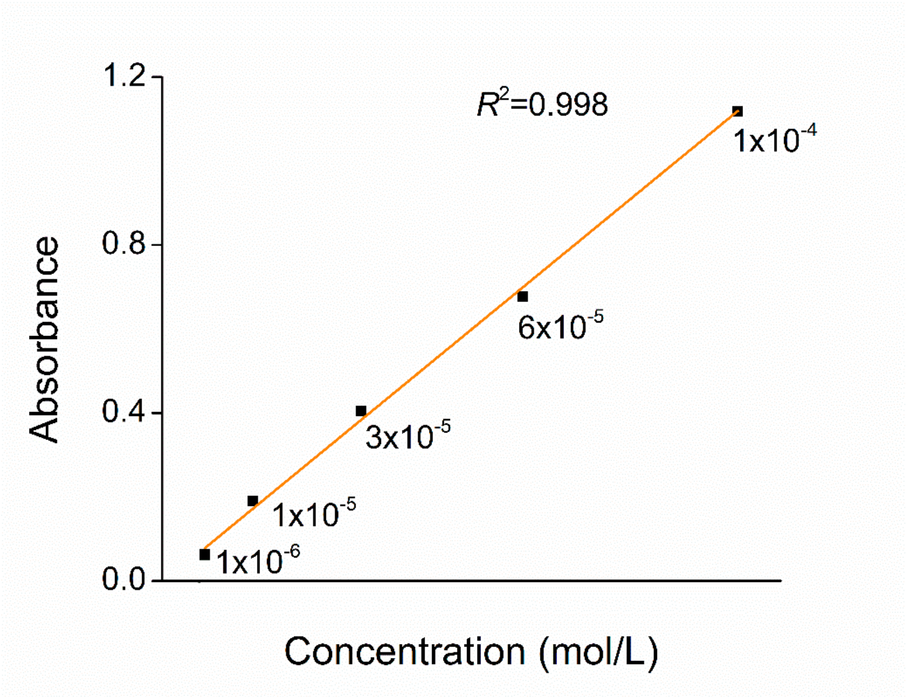

Figure S30. UV-absorption calibration curve for amine derivative **18** at the excitation wavelength of 226 nm

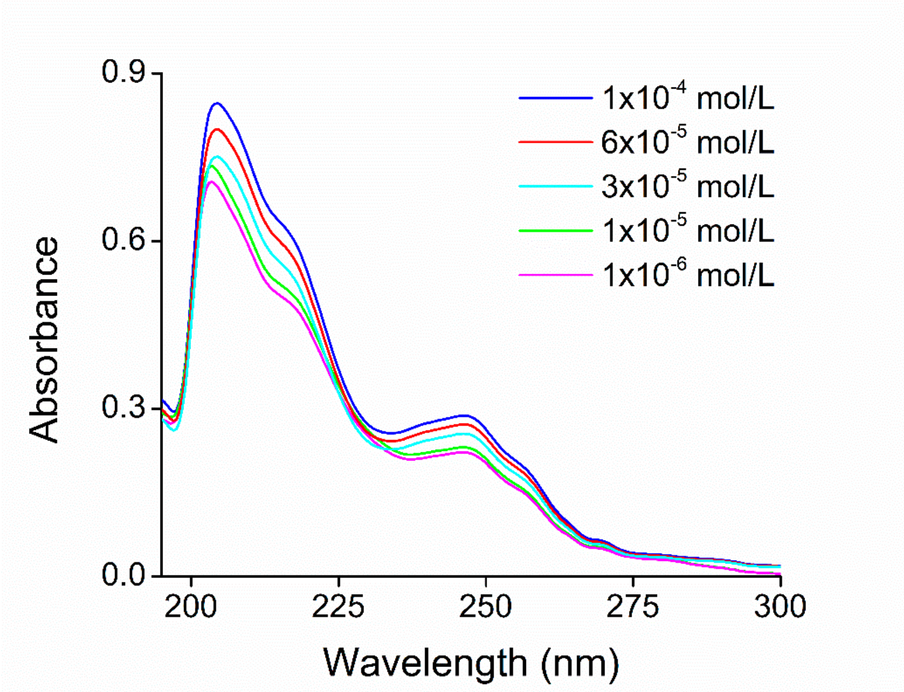

Figure S31. UV-absorption spectrum of amine derivative **19**

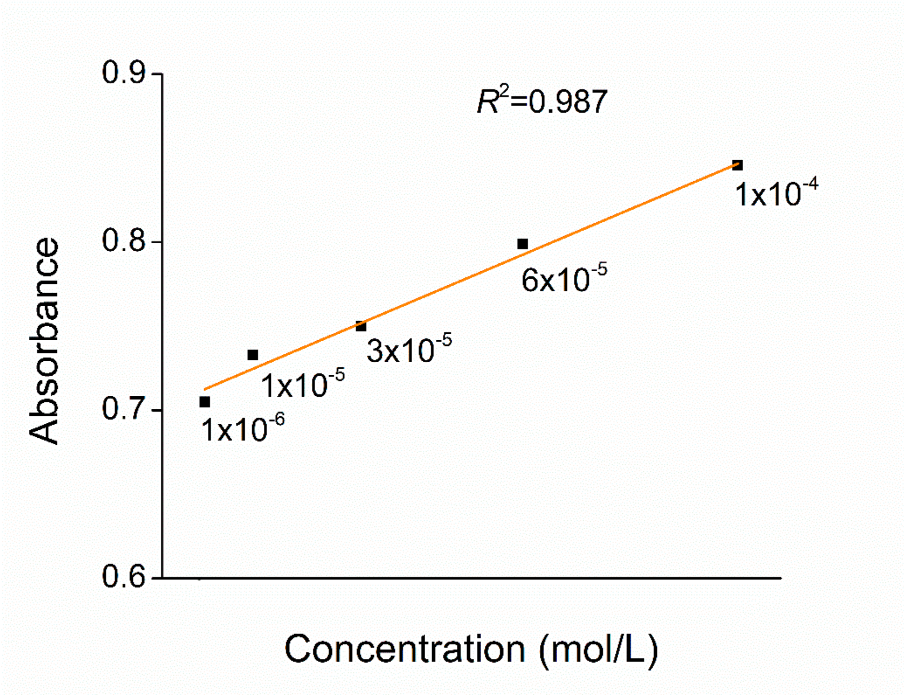

Figure S32. UV-absorption calibration curve for amine derivative **19** at the excitation wavelength of 204 nm

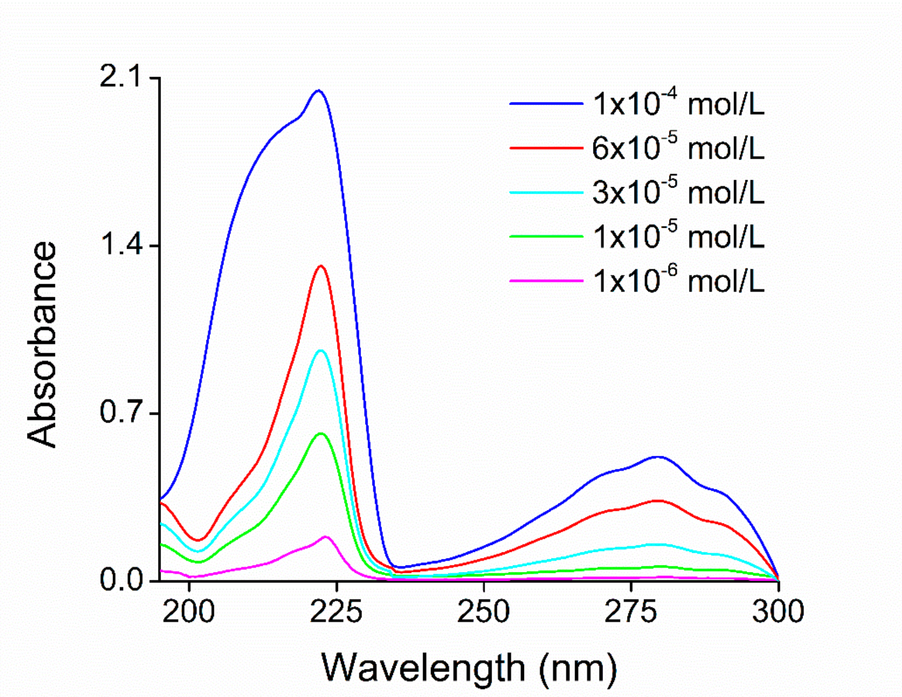

Figure S33. UV-absorption spectrum of amine derivative **20**

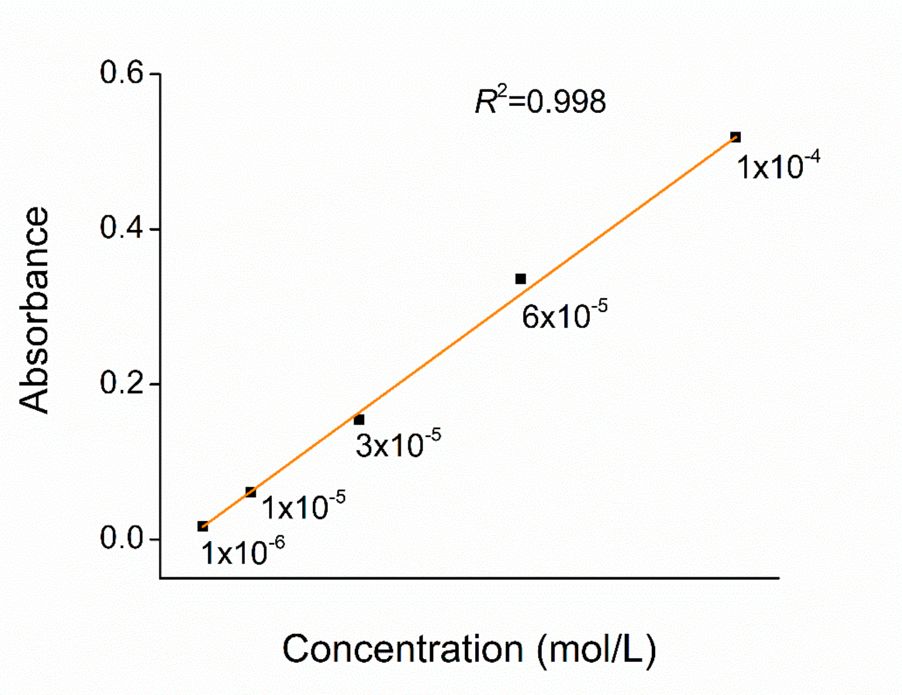

Figure S34. UV-absorption calibration curve for amine derivative **20** at the excitation wavelength of 280 nm

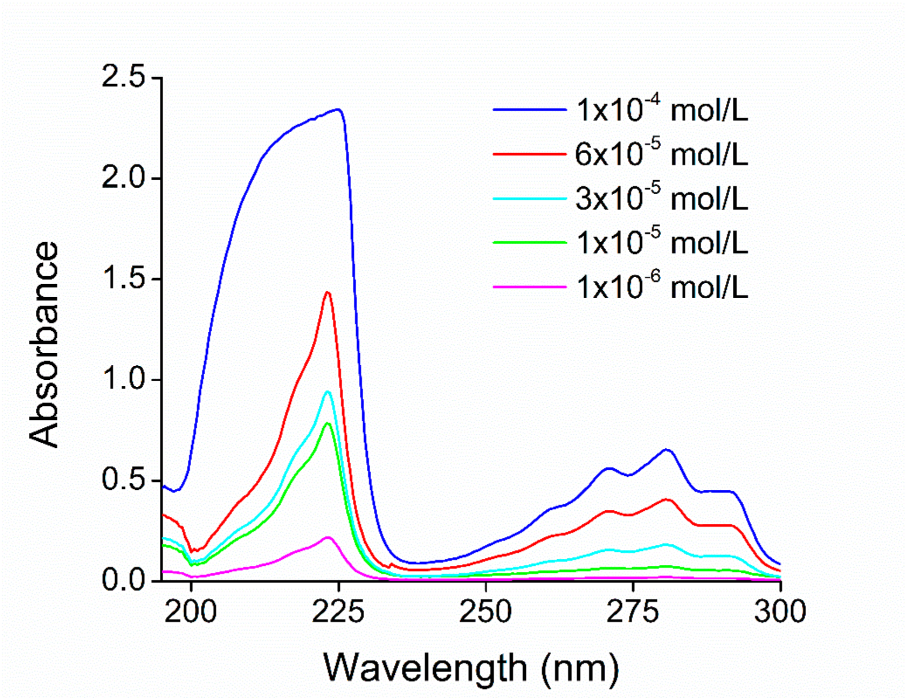

Figure S35. UV-absorption spectrum of amine derivative **21**

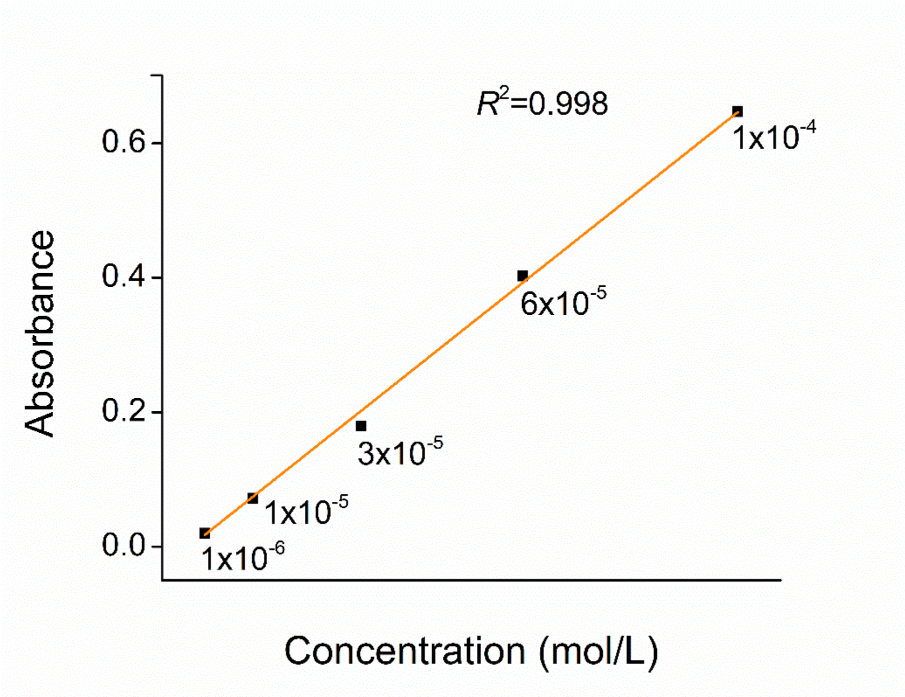

Figure S36. UV-absorption calibration curve for amine derivative **21** at the excitation wavelength of 280 nm

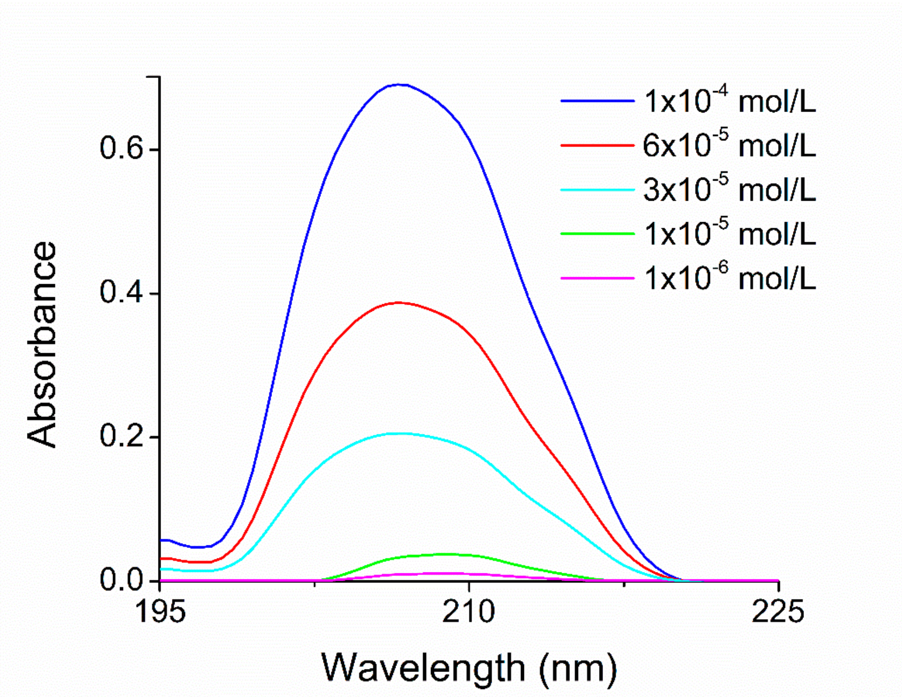

Figure S37. UV-absorption spectrum of amine derivative **22**

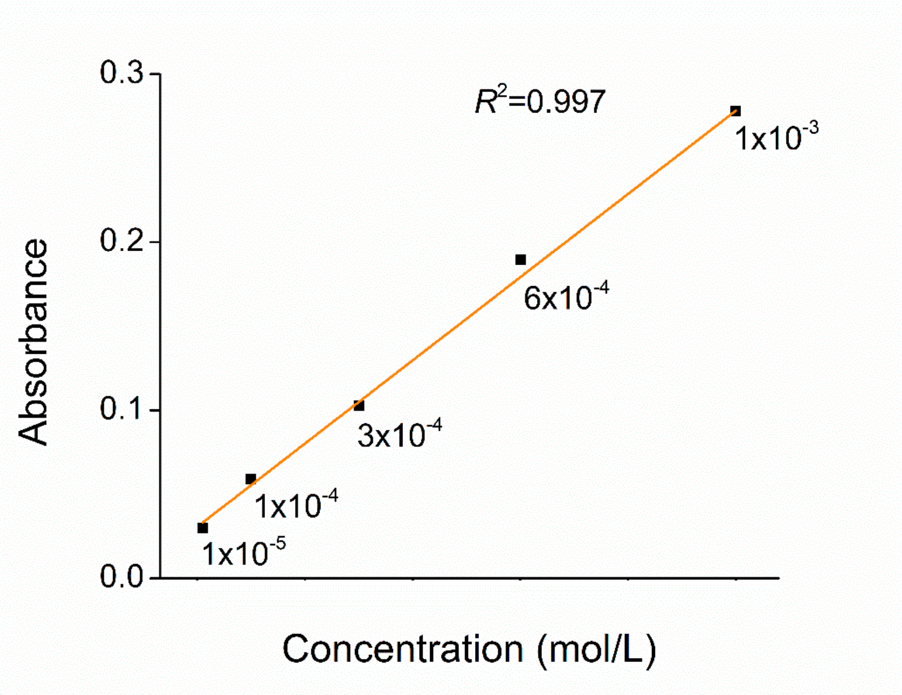

Figure S38. UV-absorption calibration curve for amine derivative **22** at the excitation wavelength of 207 nm

5. Spectrophotometric determination of complex stability constants (log*K* values) of the reported enantiopure macrocycles (*R*,*R*)-2 and (*S*,*S*)-2 with the enantiomers of various chiral protonated amine derivatives

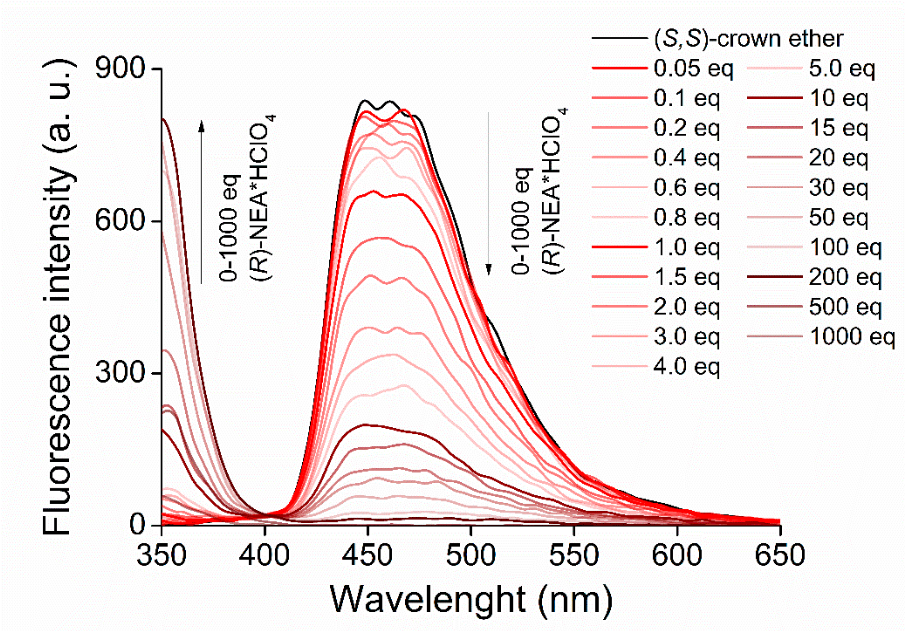

Figure S39. Fluorescence titration of macrocycle (*S*,*S*)-**2** with (*R*)-NEA (**21**)*$HClO_4$

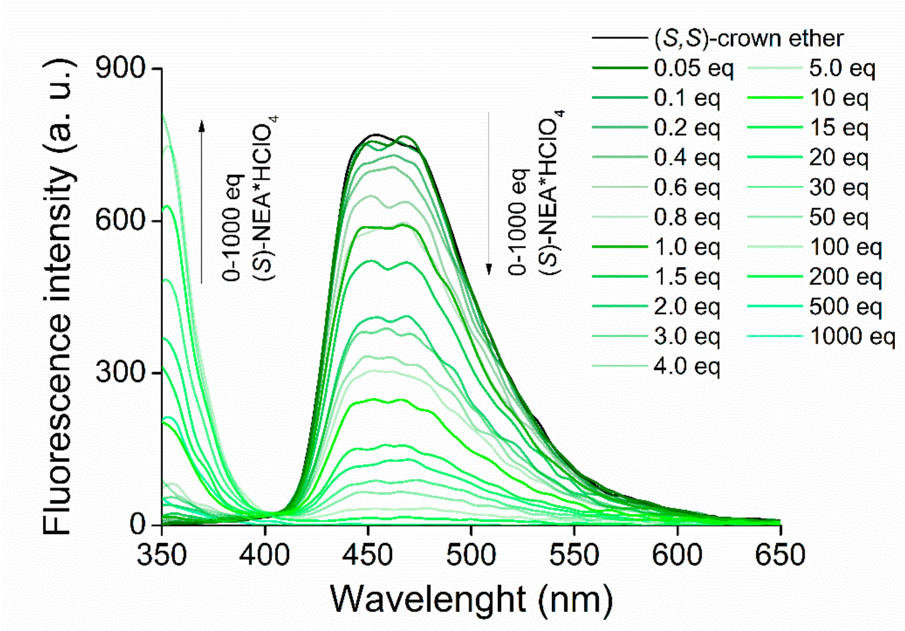

Figure S40. Fluorescence titration of macrocycle (*S*,*S*)-**2** with (*S*)-NEA (**21**)*$HClO_4$

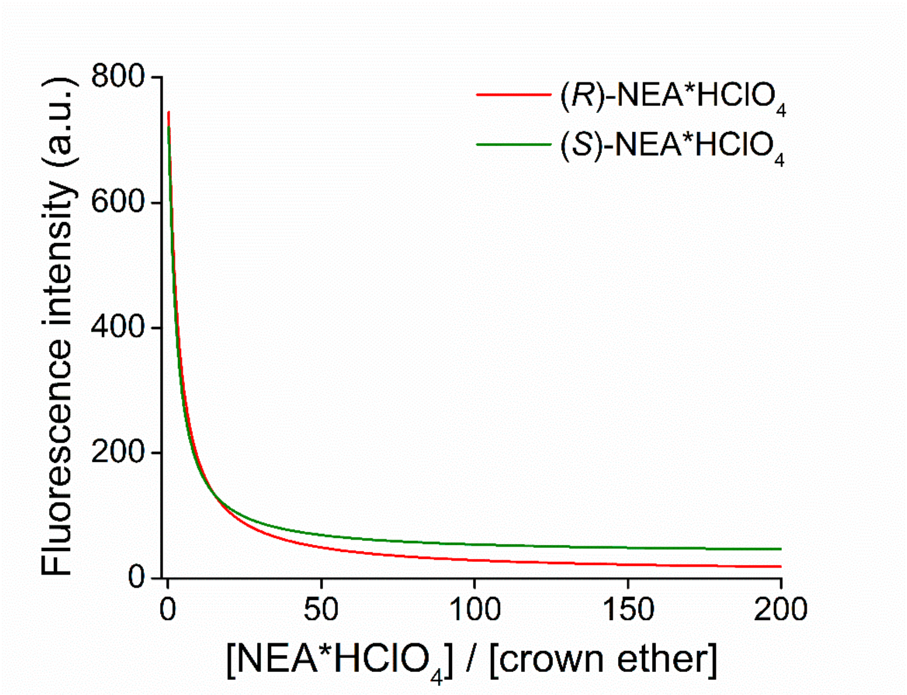

Figure S41. Fitted nonlinear functions for determining the stability constants of (*S*,*S*)-**2** with the enantiomers of NEA (**21**)*$HClO_4$

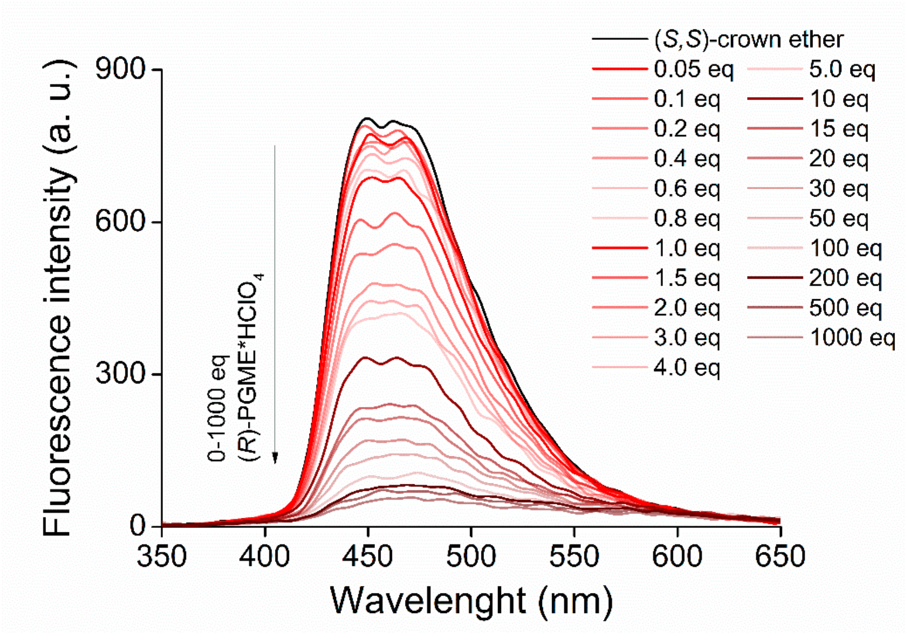

Figure S42. Fluorescence titration of macrocycle (*S*,*S*)-**2** with (*R*)-PGME (**23**)*$HClO_4$

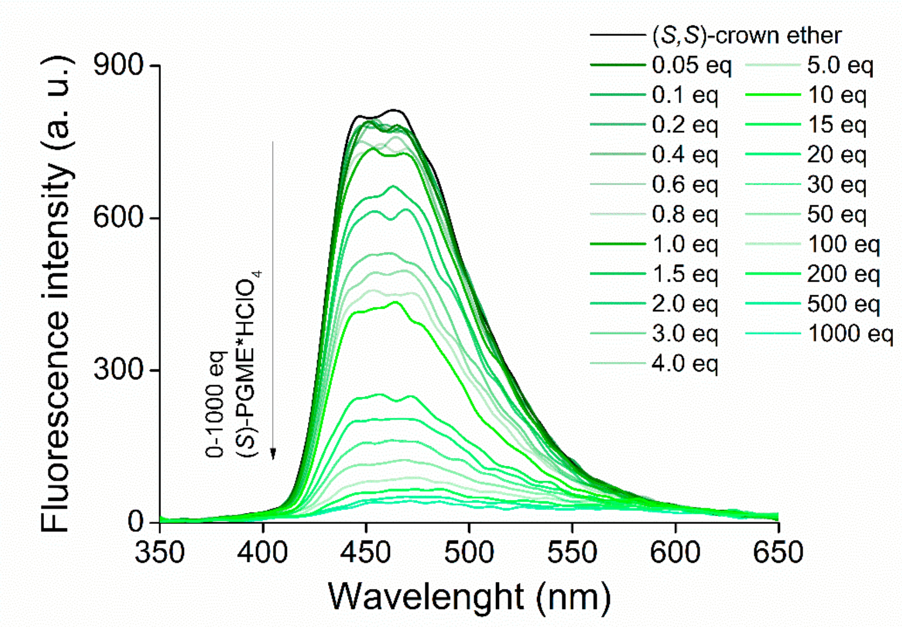

Figure S43. Fluorescence titration of macrocycle (*S*,*S*)-**2** with (*S*)-PGME (**23**)*$HClO_4$

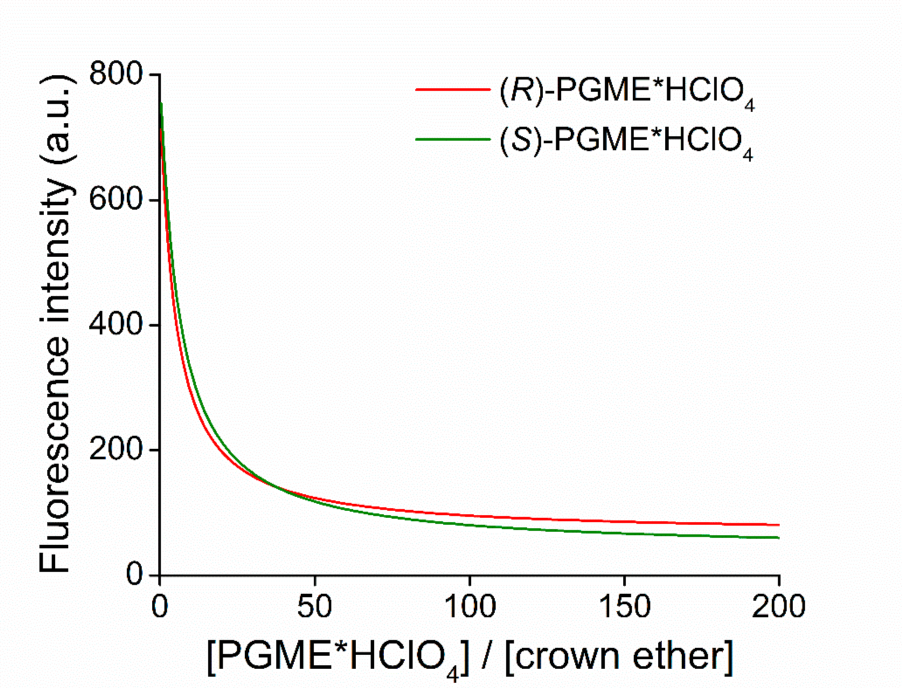

Figure S44. Fitted nonlinear functions for determining the stability constants of (*S*,*S*)-**2** with the enantiomers of PGME (**23**)*$HClO_4$

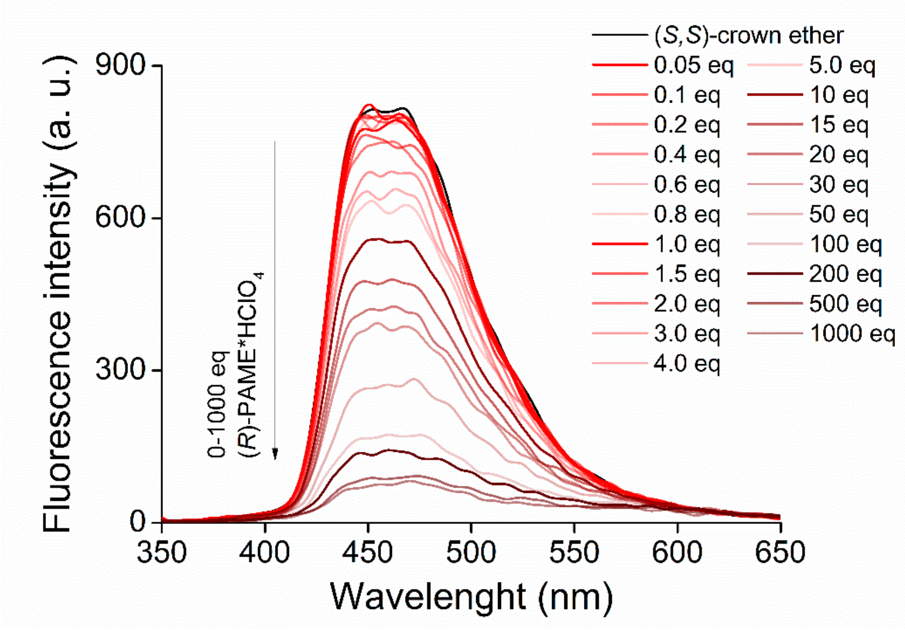

Figure S45. Fluorescence titration of macrocycle (*S*,*S*)-**2** with (*R*)-PAME (**24**)*$HClO_4$

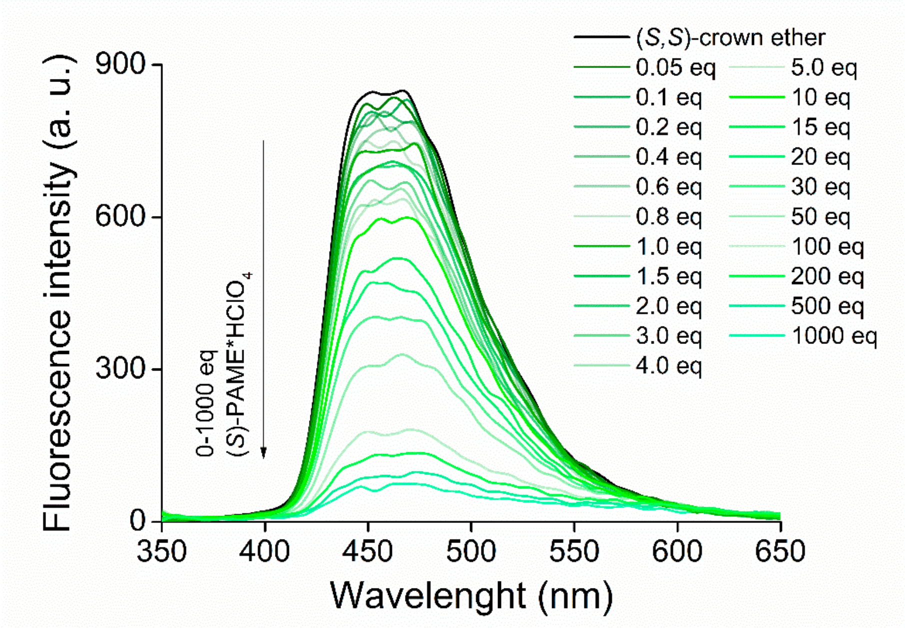

Figure S46. Fluorescence titration of macrocycle (*S*,*S*)-**2** with (*S*)-PAME (**24**)*$HClO_4$

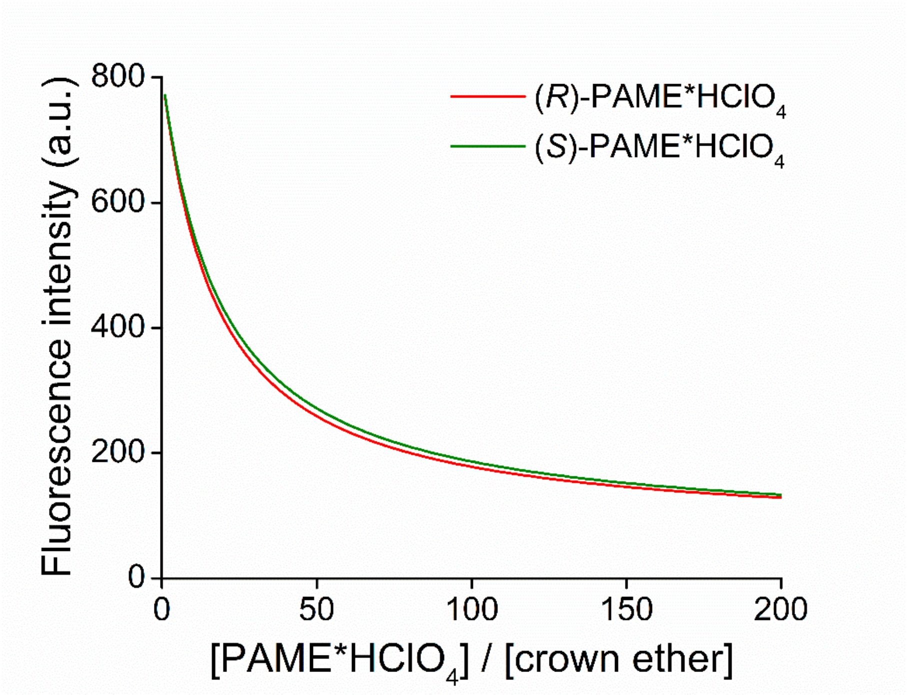

Figure S47. Fitted nonlinear functions for determining the stability constants of (*S*,*S*)-**2** with the enantiomers of PAME (**24**)*$HClO_4$

6. Electrochemical calibration curves and potentiometric selectivity measurements of ion-selective membrane electrode containing (*R*,*R*)-2 as an ionophore

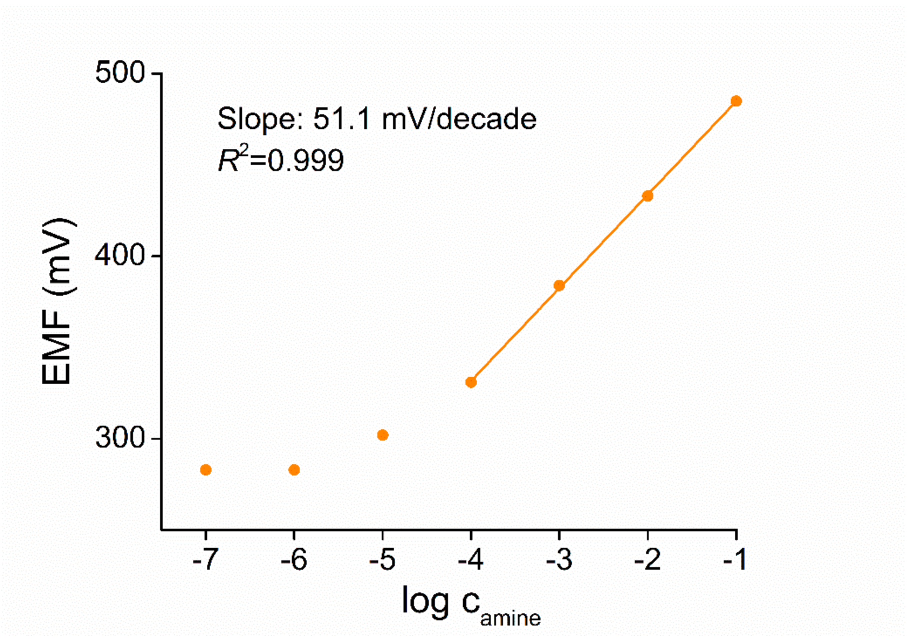

Figure S48. Calibration curve for racemic PEA (**22**)*HCl

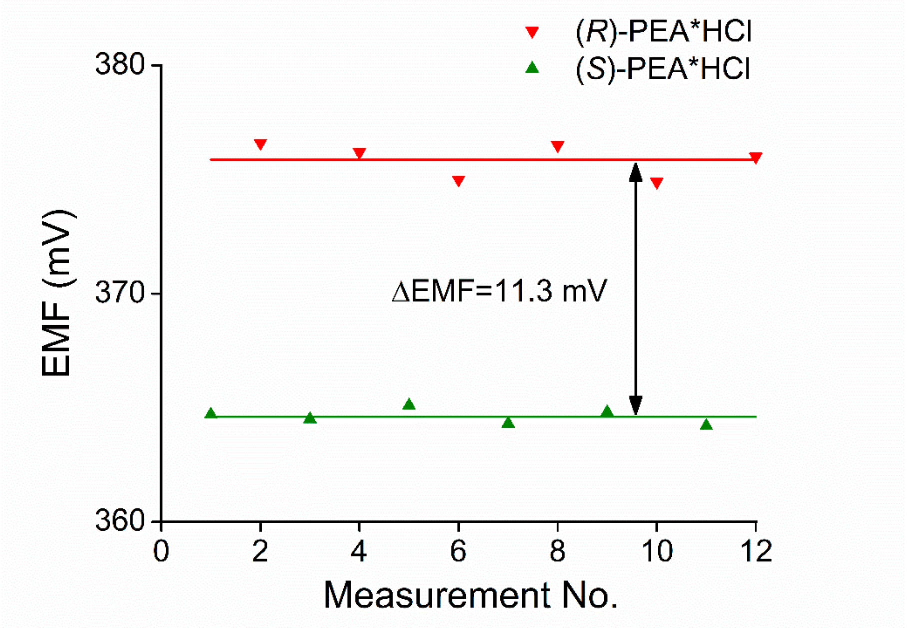

Figure S49. Measurements to determine potentiometric selectivity toward enantiomers of PEA (**22**)*HCl

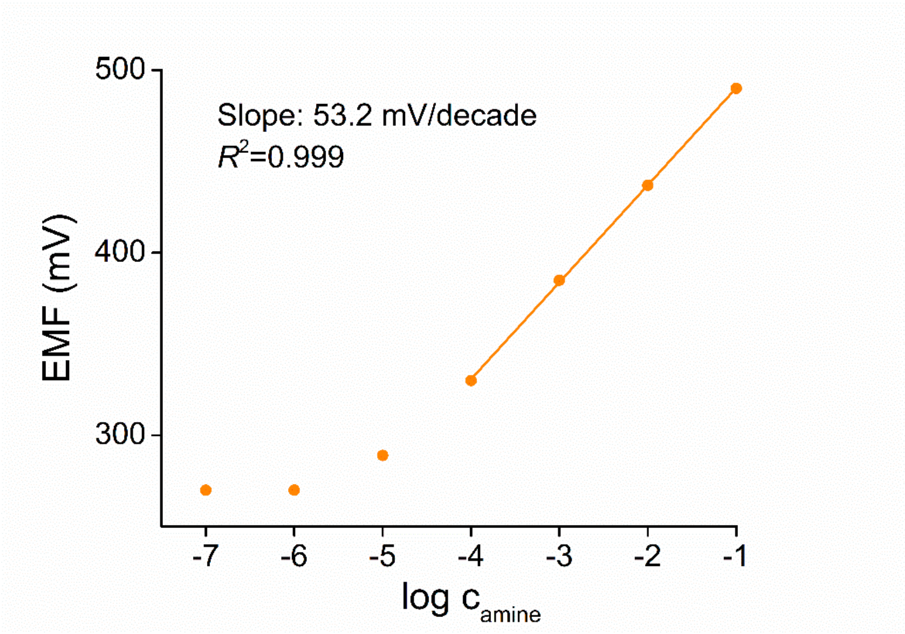

Figure S50. Calibration curve for racemic PGME (**23**)*HCl

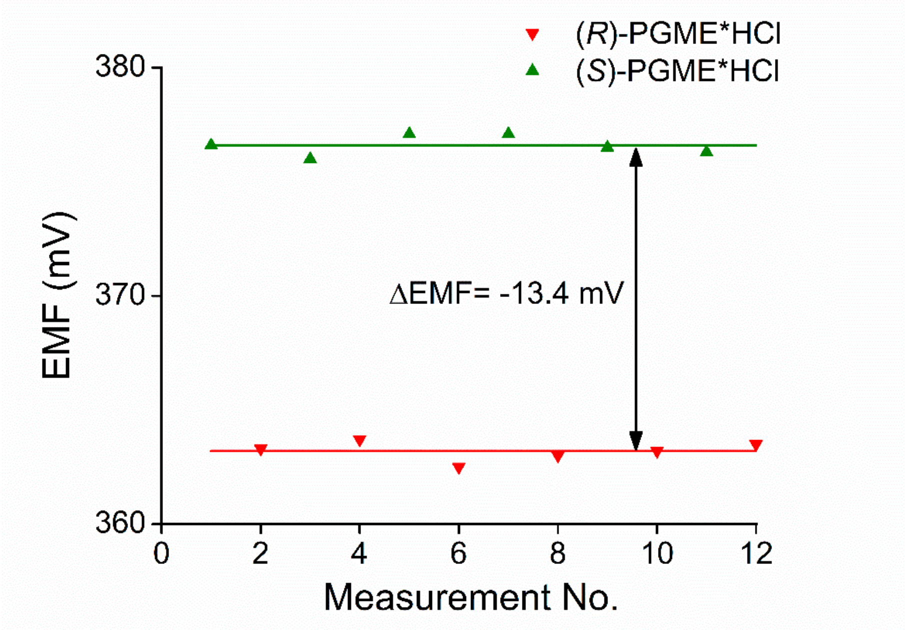

Figure S51. Measurements to determine potentiometric selectivity toward enantiomers of PGME (**23**)*HCl

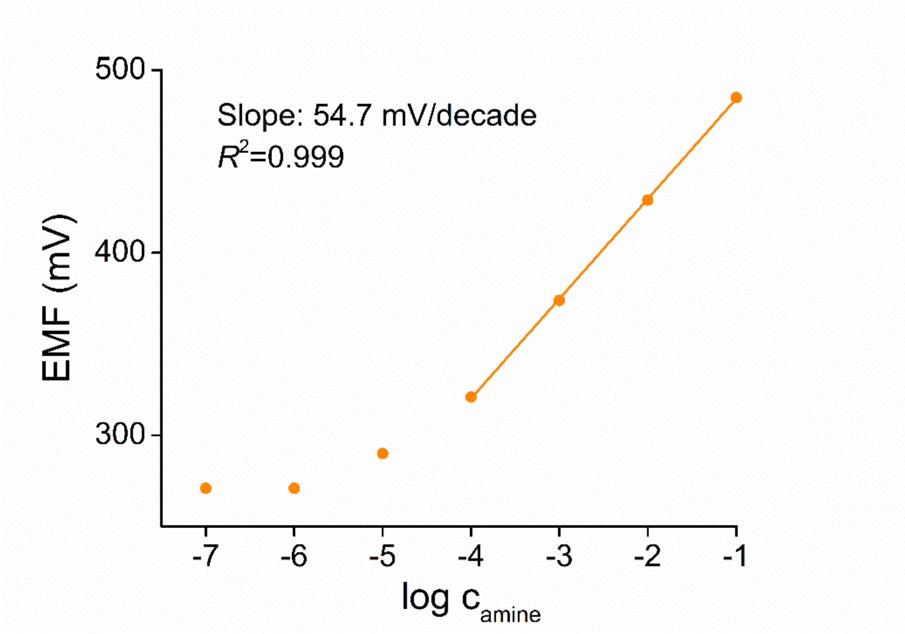

Figure S52. Calibration curve for racemic PAME (**24**)*HCl

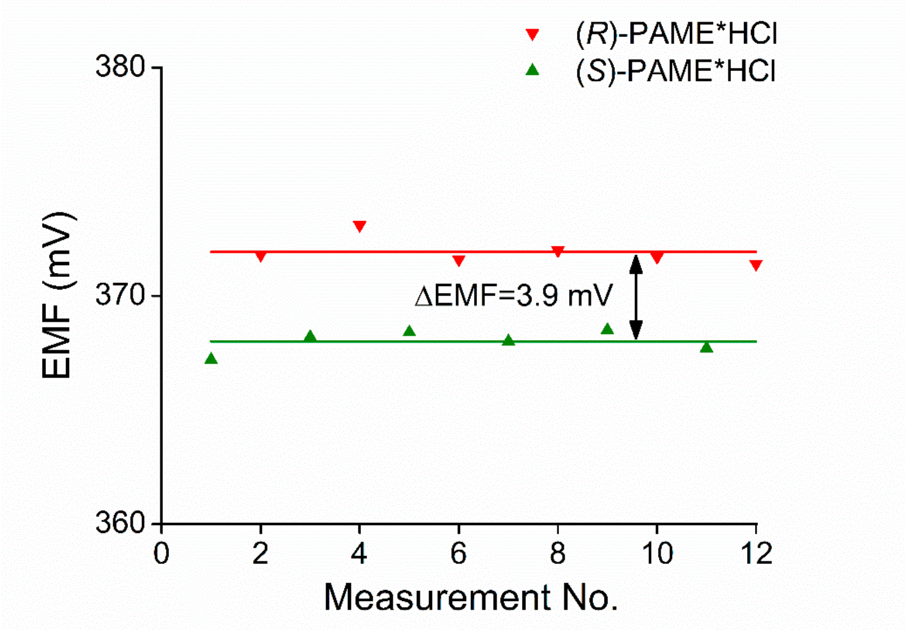

Figure S53. Measurements to determine potentiometric selectivity toward enantiomers of PAME (**24**)*HCl
